# The Real‐World Safety Profile of Haloperidol: A Comprehensive Pharmacovigilance Analysis Using the Large‐Scale FAERS Database

**DOI:** 10.1002/cns.71217

**Published:** 2026-10-20

**Authors:** Nengping Luo, Lu Xiao, Ying Liu, Xiang Liu

**Affiliations:** ^1^ Ganzhou Cancer Hospital Ganzhou Jiangxi China; ^2^ Department of Basic Medical Science Chongqing Three Gorges Medical College Chongqing China; ^3^ The Sixth People's Hospital of Chongqing The First Affiliated Hospital of Chongqing Medical and Pharmaceutical College Chongqing China

**Keywords:** adverse event, FAERS, haloperidol, pharmacovigilance, schizophrenia

## Abstract

**Objective:**

Haloperidol is a widely prescribed antipsychotic, but its long‐term safety profile across broad populations remains insufficiently researched. Using the large‐scale FDA Adverse Event Reporting System (FAERS) database, this study sought to comprehensively characterize the real‐world adverse event profile of haloperidol and identify potential safety signals.

**Methods:**

Adverse event reports from FAERS spanning Q1 2004 through Q1 2026, with haloperidol listed as the primary suspect drug, were analyzed. Disproportionality analyses employing four complementary algorithms (ROR, PRR, BCPNN, and MGPS) were conducted. Time‐to‐onset and Weibull distribution analyses were also performed.

**Results:**

A total of 9916 reports associated with haloperidol were analyzed, covering 27 System Organ Classes (SOC). The primary SOCs were nervous system disorders, psychiatric disorders, and cardiac disorders. At the preferred terms (PT) level, extrapyramidal disorder, weight increased, neuroleptic malignant syndrome, tremor, and dystonia were the most frequently reported adverse events. Cardiovascular‐related adverse events (e.g., electrocardiogram QT prolonged, tachycardia, cardiac arrest, and torsade de pointes) were confirmed. Significantly, we identified signals for some under‐recognized adverse events, including suicide‐related behaviors (intentional self‐injury, suicide attempt, suicidal ideation) and embolism venous. Most adverse events occurred within the first 30 days of treatment (72.03%), and the Weibull distribution indicated an early failure pattern, suggesting that the risk of adverse events gradually decreased over time.

**Conclusions:**

This pharmacovigilance research confirmed haloperidol's established safety profile and highlighted under‐characterized safety signals, offering valuable insight to inform clinical surveillance, risk mitigation, and future study endeavors to optimize the safe application of haloperidol across diverse patient demographics.

## Graphical abstract

This first comprehensive FAERS‐based pharmacovigilance analysis of haloperidol over two decades characterized the real‐world safety profile across 9916 reports. Strong signals were found for nervous, psychiatric, and cardiac disorders. Known risks were confirmed and novel signals revealed. Sex‐ and age‐specific vulnerabilities were identified. Findings highlight the need for enhanced clinical vigilance and dynamic monitoring.

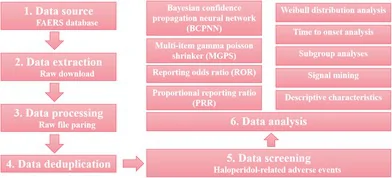

## 1 Introduction

Schizophrenia is a severe and chronic mental disorder that significantly reduces life expectancy and impairs functional ability [1]. Within the global burden of disease, schizophrenia is responsible for 15.1 million years lived with disability (YLDs) [2]. This condition causes considerable suffering, lowers quality of life, and places a heavy financial strain on healthcare systems and society [3]. The clinical presentation typically encompasses positive symptoms like hallucinations and delusions, negative symptoms such as social withdrawal and apathy, and significant cognitive impairments that are closely associated with subpar functional outcomes. Pharmacotherapy remains the foundation of management for schizophrenia spectrum disorders [4]. Antipsychotic medication is essential for managing acute psychotic episodes, preventing relapse, and maintaining long‐term clinical and functional stability [5].

Dopamine D2 receptor antagonists constitute the mainstay of pharmacotherapy for schizophrenia [6]. Since its 1958 approval by the US Food and Drug Administration, haloperidol has long served as a cornerstone among first‐generation typical antipsychotics [7]. Despite the availability of numerous more recent atypical agents, haloperidol continues to play an important role in clinical practice worldwide. In the United States alone, more than a million prescriptions are issued annually. In Canada and several areas of Europe, it continues to be one of the most often prescribed antipsychotics in long‐term care institutions and among older populations [8]. Furthermore, haloperidol is still listed on the World Health Organization's Model List of Essential Medicines because of its accessibility and low cost, which make it a useful and often required treatment choice in many developing countries. Its antipsychotic effects are mainly caused by decreased dopaminergic neurotransmission and inhibition of dopamine D2 receptors. Nevertheless, it has a poorly selective receptor binding profile and interacts with various receptor systems, which can cause a number of adverse reactions, including sedation, weight gain, and prolonged QT intervals [9, 10]. Continuous safety surveillance is necessary to fully characterize the safety profile of haloperidol over the duration of treatment, as schizophrenia usually needs extended and frequently lifetime medication.

Currently, research on haloperidol‐associated adverse events is derived predominantly from randomized controlled trials and meta‐analyses. However, these studies are frequently constrained by short follow‐up periods, extremely homogeneous patient groups, and comparatively small sample sizes, which may limit their capacity to thoroughly characterize the drug's safety profile across diverse real‐world clinical settings. Thus, real‐world pharmacovigilance research is a useful addition to conventional clinical research. In large‐scale adverse event surveillance, spontaneous reporting systems are especially significant since they offer vital data support for post‐marketing pharmacovigilance. The FDA Adverse Event Reporting System (FAERS) is one of the largest publicly available spontaneous reporting databases worldwide. It serves as a vital tool for post‐marketing pharmaceutical safety monitoring and collects real‐world safety data from a variety of demographics. In this study, we conducted a comprehensive pharmacovigilance analysis using the FAERS database to characterize the adverse event spectrum of haloperidol, identify potential safety signals, and provide evidence‐based insights to support safer prescribing practices.

## 2 Materials and Methods

### 2.1 Study Design and Data Sources

The FDA Adverse Event Reporting System (FAERS) has accumulated a substantial volume of real‐world adverse event data since 2004. All reports in which haloperidol was listed as the primary suspected drug were extracted for analysis, covering the period from the first quarter of 2004 through the first quarter of 2026. Data processing involved removing duplicate reports and standardizing adverse event terminology. Data cleaning was conducted in accordance with FDA‐recommended procedures. Specifically, for reports sharing the same case identifier (CASE_ID), the record with the most recent FDA receipt date (FDA_DT) was retained. When both CASE_ID and FDA_DT were identical, the record with the highest PRIMARY_ID was kept. The Medical Dictionary for Regulatory Activities (MedDRA) was used to standardize the coding of System Organ Class (SOC) and preferred terms (PT) signals, ensuring terminology homogeneity. Figure 1 presented a detailed flowchart of the research design.

**FIGURE 1:**
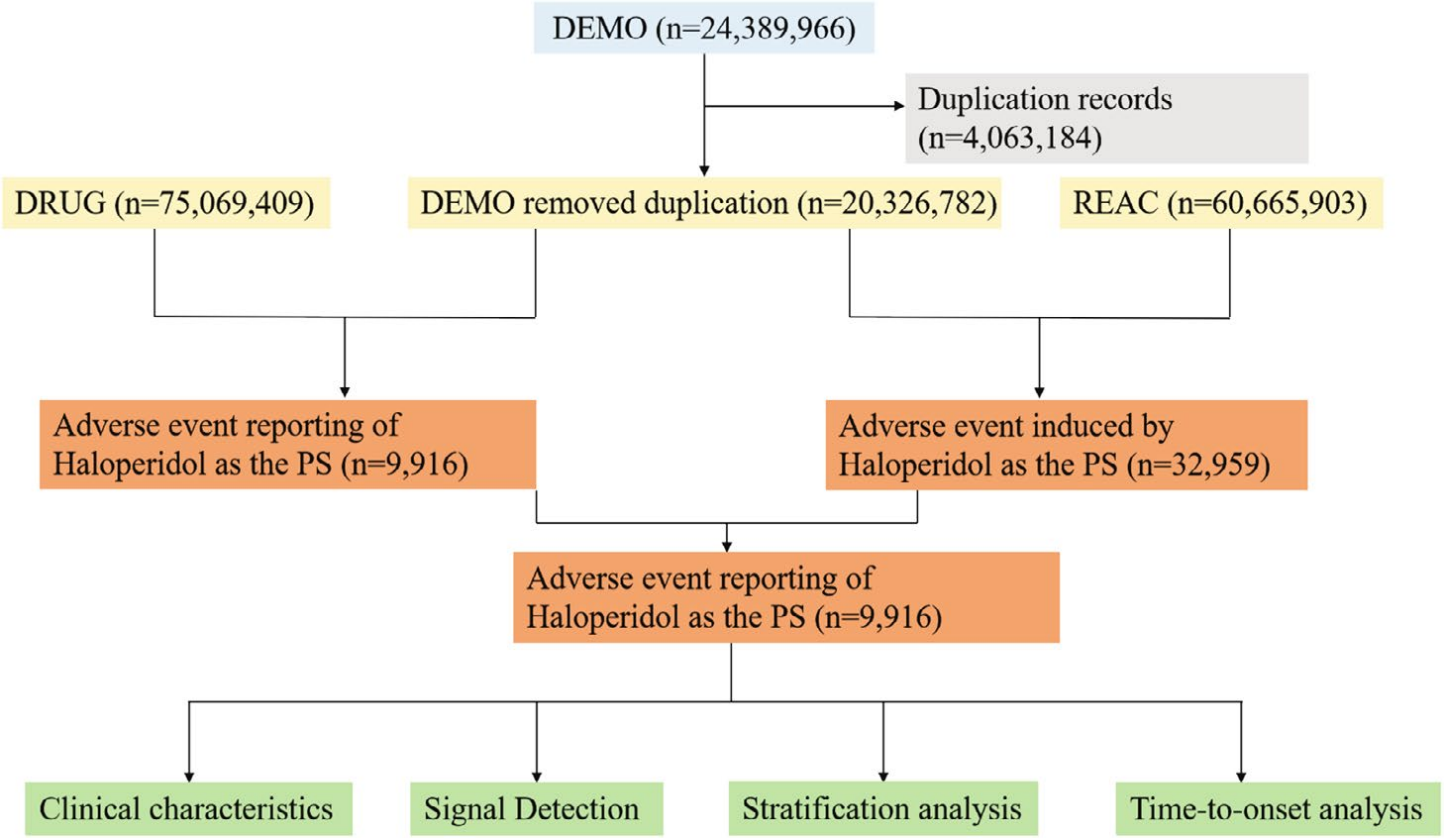
Data collection flow chart for adverse events of haloperidol. DEMO, demographic and administrative information; DRUG, drug information; PS, primary suspect; REAC, coded for the adverse events.

### 2.2 Statistical Analysis

Disproportionality analysis methods function as a fundamental pharmacovigilance tool for mining safety signals from spontaneous reporting systems, primarily aiming to identify statistically significant drug‐event associations. We used four commonly employed disproportionality analysis methods for signal detection: Bayesian Confidence Propagation Neural Network (BCPNN), Multi‐item Gamma Poisson Shrinker (MGPS), Proportional Reporting Ratio (PRR), and Reporting Odds Ratio (ROR) to guarantee the stability and reliability of findings and reduce bias from relying on a signal algorithm. ROR is used to calculate the difference in the target adverse event reporting rates between haloperidol and all other medications; it is computationally straightforward and provides intuitive results, making it particularly suitable for preliminary screening in large‐scale databases [11]. PRR calculates the relative reporting risk by comparing the proportion of a target adverse event for haloperidol to the proportion observed for all other drugs. It has a high sensitivity for identifying potential signals, but it may generate false‐positive signals when there are few reports [12]. Based on the Bayesian framework, BCPNN and MGPS are ideal for reliably identifying infrequent adverse event signals, as they reduce the impact of data sparsity and handle uncertainty more effectively than traditional frequentist approaches [13, 14]. To strengthen robustness and reduce false positive signals, especially under sparse reporting contexts, only adverse events that produced positive signals across all four algorithms were retained for further analysis. For ROR, a positive signal was defined as the lower limit of the 95% confidence interval (CI) value surpassed 1 and the number of co‐occurrences (*n*) ≥ 3; for PRR, a signal was deemed positive when the PRR value > 2, the number of co‐occurrences (*n*) ≥ 3, and variance (*χ* ^2^) > 4; for MGPS, a signal was considered positive when EBGM05 > 2; and for BCPNN, a signal was deemed positive when the lower limit of the IC025 (the lower limit of 95% CI) > 0. These thresholds were determined using standard pharmacovigilance practices. It is crucial to highlight that since FAERS lacks a reliable denominator of exposed patients, disproportionality analysis detects statistical signals rather than actual incidence rates or absolute risk. Signals identified in this study indicate statistical associations between adverse events and haloperidol. They should not be considered as measures of population‐level incidence or established causal relationships. Contingency tables and corresponding formulae for calculating signal strength thresholds using ROR, PRR, BCPNN, and MGPS were provided in Tables S1 and S2. The higher the value, the stronger the signal, suggesting a greater association between the adverse event and haloperidol. R version 4.4 was used for all statistical analyses.

### 2.3 Time‐to‐Onset and Weibull Distribution Analysis

Weibull distribution and time‐to‐onset (TTO) analysis were performed to assess the temporal risk dynamics of adverse events related to haloperidol. The interval between the haloperidol start date (START_DT) and the adverse event onset time (EVENT_DT) was used to calculate the TTO. Reports with missing dates, negative onset times caused by EVENT_DT being earlier than START_DT, or improbable onset timings (e.g., surpassing the study period) were eliminated. These standards guaranteed that the data used in our study were accurate and reliable.

The Weibull distribution is characterized by two important parameters: the scale parameter (*α*) and shape parameter (*β*). The distribution's temporal concentration is reflected in the scale parameter (*α*), where smaller values indicate a more concentrated onset pattern and bigger values indicate more dispersion over time. The dynamic variation in hazard over time is indicated by the shape parameter (*β*): an early failure type is characterized by a declining risk rate with time when the upper limit of the 95% confidence interval (CI) of *β* is less than 1. The risk rate is constant when the upper limit of the 95% confidence interval (CI) of *β* is close to 1, and its 95% confidence interval includes 1, which is indicative of a random failure type. Finally, when the lower limit of the 95% CI for *β* surpasses 1, the risk rate grows with time, suggesting a wear‐out failure type.

## 3 Results

### 3.1 Clinical Characteristics Description

A total of 9916 cases were identified in which haloperidol was listed as the primary suspected drug, corresponding to 32,959 adverse event records. The baseline characteristics of these cases were presented in Table 1. Reports from males constituted a larger proportion (49.2%, *n* = 4879) compared to those from females (37.2%, *n* = 3692). The majority of cases (50.2%, *n* = 4982) occurred in patients aged 18–65 years, followed by those over 65 years (14.6%, *n* = 1452). Serious outcomes were documented in over half of the cases (62.7%, *n* = 6215). Geographically, the United States contributed the largest share of reports (42.6%, *n* = 4198), with Italy (10.2%, *n* = 1008) and France (9.8%, *n* = 966) being the next most frequent contributors. Most reports originated from healthcare professionals (55.3%, *n* = 5483), followed by consumers (22.4%, *n* = 2220).

**TABLE 1:** Clinical characteristics of haloperidol adverse event reports from the FAERS database (Q1 2004 to Q1 2026).

Characteristics	Case numbers	Case proportion (%)
Number	9916	
Gender		
Female	3692	37.2%
Male	4879	49.2%
Miss	1345	13.6%
Age		
< 18	450	4.5%
18–65	4982	50.2%
> 65	1452	14.6%
Miss	3032	30.6%
Top 5 reported countries		
United States	4198	42.6%
Italy	1008	10.2%
France	966	9.8%
United Kingdom	639	6.5%
Germany	444	4.5%
Reporter		
Healthcare professional	5483	55.3%
Consumer	2220	22.4%
Miss	2213	22.3%
Serious cases		
No	3701	37.3%
Yes	6215	62.7%
Reported years		
2004	61	0.60%
2005	80	0.80%
2006	96	1.00%
2007	860	8.70%
2008	88	0.90%
2009	240	2.40%
2010	203	2.10%
2011	222	2.30%
2012	261	2.70%
2013	251	2.50%
2014	416	4.20%
2015	995	10.10%
2016	850	8.60%
2017	766	7.80%
2018	770	7.80%
2019	630	6.40%
2020	669	6.80%
2021	447	4.50%
2022	604	6.10%
2023	576	5.80%
2024	351	3.60%
2025	412	4.20%
Q1 2026	68	0.69%

### 3.2 Signal Mining Results

The system organ class (SOC) level signals associated with haloperidol were presented in Table 2. A total of 27 SOCs were identified (Figure 2). The most commonly reported SOCs were Nervous system disorders, Psychiatric disorders, and General disorders and administration site conditions. The strongest signals were found in Psychiatric disorders, Nervous system disorders, and Endocrine disorders. Other SOCs, such as Cardiac disorders and Immune system disorders, also showed robust signals. These findings suggested that increased clinical vigilance is warranted for psychiatric, neurological, and cardiac adverse reactions associated with haloperidol.

**TABLE 2:** Signal of haloperidol adverse events across System Organ Classes (SOC) in the FAERS database.

SOC	Case numbers	ROR (95% Cl)	PRR (c2)	EBGM (EBGM05)	IC (IC025)
Eye disorders	657	0.68 (0.63–0.74)	0.69 (94.75)	0.69 (0.64)	−0.54 (−0.65)
Investigations	3232	1.13 (1.09–1.17)	1.12 (43.48)	1.12 (1.08)	0.16 (0.11)
Product issues	389	0.49 (0.44–0.54)	0.49 (210.53)	0.49 (0.44)	−1.03 (−1.18)
Endocrine disorders	227	1.85 (1.62–2.11)	1.84 (87.86)	1.84 (1.62)	0.88 (0.69)
Vascular disorders	838	0.83 (0.78–0.89)	0.84 (27.71)	0.84 (0.78)	−0.26 (−0.36)
Social circumstances	317	1.43 (1.28–1.6)	1.43 (41.29)	1.43 (1.28)	0.52 (0.35)
Hepatobiliary disorders	220	0.51 (0.45–0.58)	0.51 (102.01)	0.51 (0.45)	−0.96 (−1.15)
Nervous System Disorders	10,309	3 (2.93–3.06)	2.57 (10746.06)	2.56 (2.51)	1.36 (1.33)
Immune system disorders	662	1.2 (1.12–1.3)	1.2 (22.71)	1.2 (1.11)	0.27 (0.15)
Cardiac disorders	1708	1.43 (1.36–1.5)	1.41 (212.21)	1.41 (1.35)	0.5 (0.43)
Psychiatric disorders	7372	3.16 (3.08–3.24)	2.83 (9186.25)	2.82 (2.75)	1.5 (1.46)
Musculoskeletal and connective tissue disorders	1966	0.78 (0.75–0.82)	0.79 (117.49)	0.79 (0.75)	−0.34 (−0.41)
Congenital, familial and genetic disorders	170	1.2 (1.03–1.4)	1.2 (5.75)	1.2 (1.03)	0.26 (0.04)
Neoplasms benign, malignant and unspecified (including cysts and polyps)	119	0.1 (0.09–0.12)	0.1 (932.25)	0.1 (0.09)	−3.25 (−3.51)
Metabolism and nutrition disorders	1040	1 (0.94–1.07)	1 (0.01)	1 (0.94)	0 (−0.09)
Reproductive system and breast disorders	302	0.7 (0.63–0.79)	0.71 (37.47)	0.71 (0.63)	−0.5 (−0.67)
Surgical and medical procedures	449	0.69 (0.63–0.76)	0.69 (62.29)	0.69 (0.63)	−0.53 (−0.67)
General disorders and administration site conditions	5976	0.67 (0.65–0.69)	0.71 (850.72)	0.71 (0.69)	−0.49 (−0.53)
Injury, poisoning and procedural complications	5328	1.05 (1.03–1.09)	1.05 (13.48)	1.05 (1.02)	0.07 (0.03)
Blood and lymphatic system disorders	514	0.65 (0.59–0.7)	0.65 (99.28)	0.65 (0.59)	−0.62 (−0.75)
Pregnancy, puerperium and perinatal conditions	120	0.59 (0.49–0.71)	0.59 (33.69)	0.59 (0.5)	−0.75 (−1.01)
Infections and infestations	801	0.31 (0.29–0.33)	0.32 (1230.91)	0.32 (0.3)	−1.65 (−1.75)
Gastrointestinal disorders	2106	0.49 (0.47–0.51)	0.51 (1058.3)	0.51 (0.49)	−0.96 (−1.02)
Ear and labyrinth disorders	80	0.38 (0.3–0.47)	0.38 (81.61)	0.38 (0.3)	−1.4 (−1.71)
Renal and urinary disorders	685	0.73 (0.68–0.79)	0.74 (65.96)	0.74 (0.68)	−0.44 (−0.55)
Skin and subcutaneous tissue disorders	855	0.31 (0.29–0.33)	0.32 (1297.93)	0.32 (0.3)	−1.64 (−1.74)
Respiratory, thoracic and mediastinal disorders	1384	0.6 (0.57–0.64)	0.61 (351.16)	0.61 (0.58)	−0.7 (−0.78)

Abbreviations: CI, confidence interval; EBGM, empirical Bayesian geometric mean; EBGM05, the lower limit of the 95% CI of EBGM; IC, information component; IC025, the lower limit of the 95% CI of the IC; PRR, proportional reporting ratio; ROR, reporting odds ratio; SOC, System Organ Classes.

**FIGURE 2:**
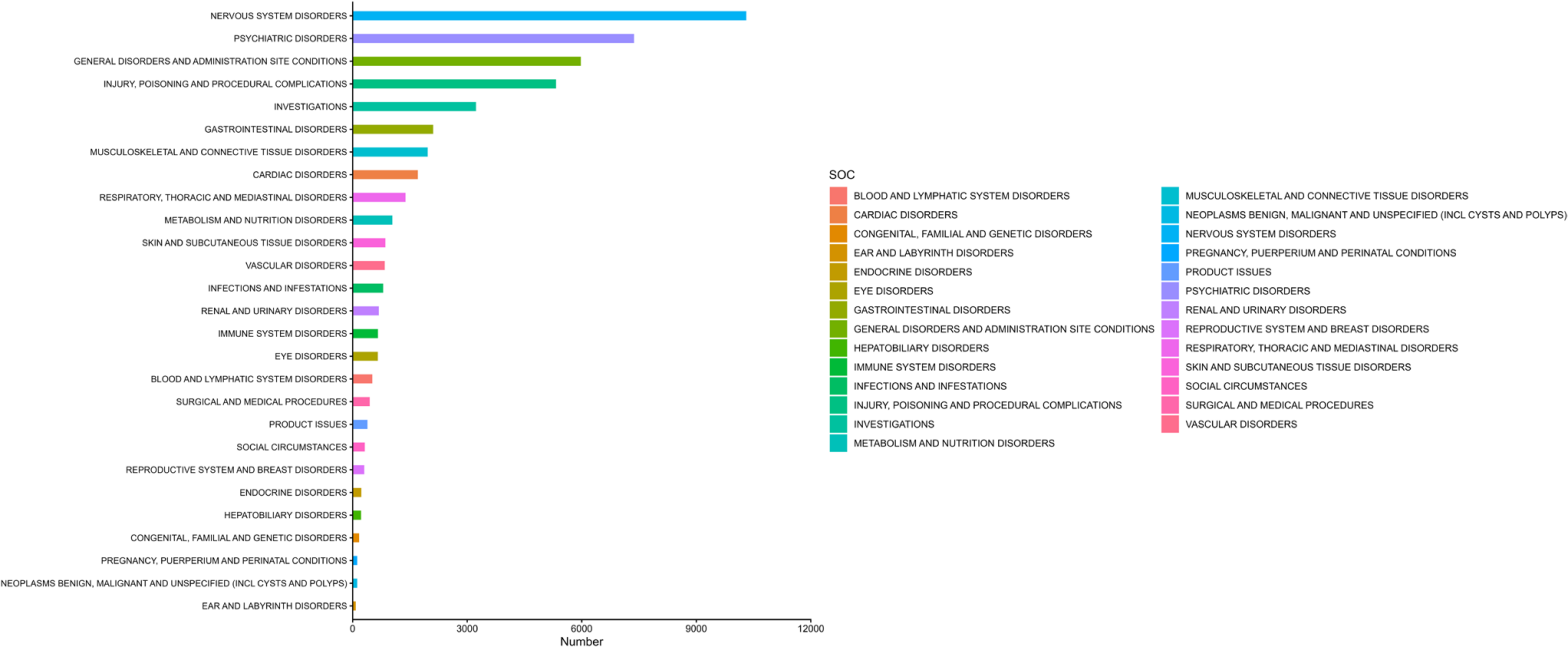
System Organ Classes (SOC) distribution.

At the Preferred Term (PT) level, all haloperidol‐associated adverse events were systematically ranked and evaluated. The 50 most frequently reported PTs were listed in Table 3, with their visualization presented in Figure 3. The top 50 PTs ranked by ROR signal strength were displayed in Figure 4. The top 10 most commonly reported PTs were extrapyramidal disorder, weight increased, neuroleptic malignant syndrome, tremor, dystonia, drug interaction, somnolence, agitation, and intentional self‐injury. Except for weight increased, agitation, and intentional self‐injury, these findings were consistent with the adverse effects described in the prescribing information. The PTs exhibiting the strongest signal strengths included neuroleptic‐induced deficit syndrome, malignant catatonia, pharyngeal dystonia, spasmodic dysphonia, oromandibular dystonia, neuroleptic malignant syndrome, cogwheel rigidity, protrusion tongue, osteochondritis, and catatonia. Furthermore, several potential adverse reactions not currently listed in the drug label were identified, including delirium, rhabdomyolysis, blood creatine phosphokinase increased, cardiac arrest, suicidal ideation, dysarthria, self‐destructive behavior, embolism venous, and auditory hallucination.

**TABLE 3:** Top 50 frequency of adverse events at the PT level for haloperidol.

PT	Case numbers	ROR (95% CI)	PRR (*χ* ^2^)	EBGM (EBGM05)	IC (IC025)
Extrapyramidal disorder	1072	67.05 (63.04–71.31)	65.57 (65688.07)	63.2 (59.42)	5.98 (5.81)
Weight increased^a^	936	5.54 (5.19–5.91)	5.45 (3404.3)	5.44 (5.1)	2.44 (2.34)
Neuroleptic malignant syndrome	814	96.96 (90.3–104.11)	95.33 (72015.55)	90.39 (84.18)	6.5 (6.24)
Tremor	593	4.63 (4.27–5.02)	4.58 (1662.03)	4.57 (4.22)	2.19 (2.07)
Dystonia	576	44.65 (41.09–48.53)	44.13 (23679.52)	43.05 (39.62)	5.43 (5.2)
Drug interaction	574	4.73 (4.36–5.14)	4.69 (1664.72)	4.68 (4.31)	2.23 (2.1)
Somnolence	543	3.49 (3.2–3.8)	3.46 (950.34)	3.45 (3.17)	1.79 (1.66)
Agitation^a^	419	7.34 (6.67–8.09)	7.29 (2266.85)	7.26 (6.6)	2.86 (2.7)
Intentional self‐injury^a^	402	23.34 (21.14–25.76)	23.15 (8409.41)	22.86 (20.7)	4.51 (4.29)
Drug hypersensitivity^a^	390	2.3 (2.08–2.54)	2.29 (284)	2.29 (2.07)	1.19 (1.04)
Electrocardiogram QT prolonged	356	12.82 (11.55–14.23)	12.73 (3822.61)	12.65 (11.39)	3.66 (3.46)
Intentional overdose^a^	353	6.96 (6.27–7.73)	6.92 (1781.17)	6.89 (6.21)	2.79 (2.61)
Akathisia	334	33.19 (29.77–37)	32.96 (10159.88)	32.36 (29.03)	5.02 (4.73)
Dyskinesia	332	11.02 (9.89–12.28)	10.95 (2983.8)	10.88 (9.77)	3.44 (3.24)
Confusional state	319	2.54 (2.27–2.84)	2.53 (295.37)	2.53 (2.26)	1.34 (1.17)
Muscle rigidity	310	34.79 (31.08–38.94)	34.57 (9908.2)	33.91 (30.29)	5.08 (4.77)
Tardive dyskinesia	287	15.75 (14.01–17.69)	15.66 (3904.2)	15.53 (13.82)	3.96 (3.71)
Tachycardia	278	4.16 (3.69–4.68)	4.14 (660.87)	4.13 (3.67)	2.05 (1.86)
Dysphagia	277	3.67 (3.26–4.13)	3.65 (532.71)	3.64 (3.24)	1.87 (1.68)
Musculoskeletal stiffness	262	3.84 (3.4–4.34)	3.83 (546.73)	3.82 (3.38)	1.93 (1.74)
Sopor	261	22.39 (19.81–25.31)	22.28 (5237.46)	22 (19.47)	4.46 (4.17)
Catatonia	260	68.59 (60.57–77.66)	68.22 (16567.96)	65.67 (57.99)	6.04 (5.54)
Parkinsonism^a^	255	36.41 (32.16–41.24)	36.22 (8556.09)	35.5 (31.35)	5.15 (4.78)
Aggression^a^	255	6.69 (5.91–7.57)	6.66 (1222.55)	6.64 (5.87)	2.73 (2.52)
Drug abuse^a^	255	3.85 (3.4–4.35)	3.83 (532.83)	3.82 (3.38)	1.94 (1.74)
Delirium^a^	250	9.71 (8.58–11)	9.67 (1933.13)	9.62 (8.49)	3.27 (3.03)
Psychotic disorder	246	11.12 (9.8–12.61)	11.07 (2239.13)	11 (9.7)	3.46 (3.22)
Sedation	244	12.84 (11.32–14.57)	12.78 (2630.85)	12.69 (11.19)	3.67 (3.41)
Suicide attempt^a^	244	5.41 (4.77–6.14)	5.39 (869.74)	5.37 (4.74)	2.43 (2.22)
Coma	237	6.55 (5.76–7.44)	6.52 (1104.23)	6.5 (5.72)	2.7 (2.48)
Hallucination	222	3.98 (3.49–4.55)	3.97 (492.53)	3.96 (3.47)	1.99 (1.77)
Rhabdomyolysis^a^	217	7.16 (6.26–8.18)	7.13 (1139.37)	7.1 (6.21)	2.83 (2.59)
Cognitive disorder^a^	212	5.91 (5.16–6.76)	5.88 (857.16)	5.87 (5.13)	2.55 (2.32)
Restlessness	193	6.83 (5.93–7.87)	6.81 (953.04)	6.78 (5.89)	2.76 (2.51)
Blood creatine phosphokinase increased^a^	186	8.65 (7.48–9.99)	8.62 (1246.4)	8.58 (7.42)	3.1 (2.83)
Cardiac arrest^a^	181	2.9 (2.51–3.36)	2.89 (224.36)	2.89 (2.5)	1.53 (1.3)
Abnormal behavior	180	5.82 (5.03–6.74)	5.8 (713.45)	5.79 (5)	2.53 (2.28)
Speech disorder	180	4.44 (3.84–5.14)	4.43 (476.87)	4.42 (3.82)	2.14 (1.9)
Suicidal ideation^a^	179	2.56 (2.21–2.96)	2.55 (168.84)	2.55 (2.2)	1.35 (1.12)
Schizophrenia^a^	171	18.09 (15.56–21.04)	18.03 (2723.23)	17.86 (15.36)	4.16 (3.8)
Dysarthria^a^	165	5.76 (4.94–6.71)	5.74 (644.3)	5.73 (4.91)	2.52 (2.25)
Self‐destructive behavior^a^	158	62.88 (53.64–73.72)	62.68 (9254)	60.52 (51.62)	5.92 (5.23)
Depressed level of consciousness	156	5.35 (4.57–6.26)	5.33 (547.57)	5.32 (4.54)	2.41 (2.14)
Salivary hypersecretion	143	18.92 (16.04–22.32)	18.87 (2393.99)	18.68 (15.83)	4.22 (3.81)
Embolism venous^a^	140	59.93 (50.62–70.94)	59.75 (7817.51)	57.79 (48.81)	5.85 (5.12)
Hypothermia^a^	138	17.64 (14.91–20.87)	17.59 (2138.38)	17.43 (14.73)	4.12 (3.72)
Hallucination, auditory^a^	136	11.44 (9.67–13.55)	11.41 (1284.17)	11.35 (9.58)	3.5 (3.15)
Torsade de pointes	135	23.23 (19.6–27.54)	23.17 (2826.06)	22.88 (19.3)	4.52 (4.05)
Hypertonia	134	32.65 (27.51–38.74)	32.56 (4023.81)	31.98 (26.95)	5 (4.45)
Drooling	133	26.08 (21.97–30.96)	26.01 (3151.6)	25.64 (21.6)	4.68 (4.19)

Abbreviations: CI, confidence interval; EBGM, empirical Bayesian geometric mean; EBGM05, the lower limit of the 95% CI of EBGM; IC, information component; IC025, the lower limit of the 95% CI of the IC; PRR, proportional reporting ratio; PT, preferred term; ROR, reporting odds ratio.

^a^ For adverse events not documented in the instructions.

**FIGURE 3:**
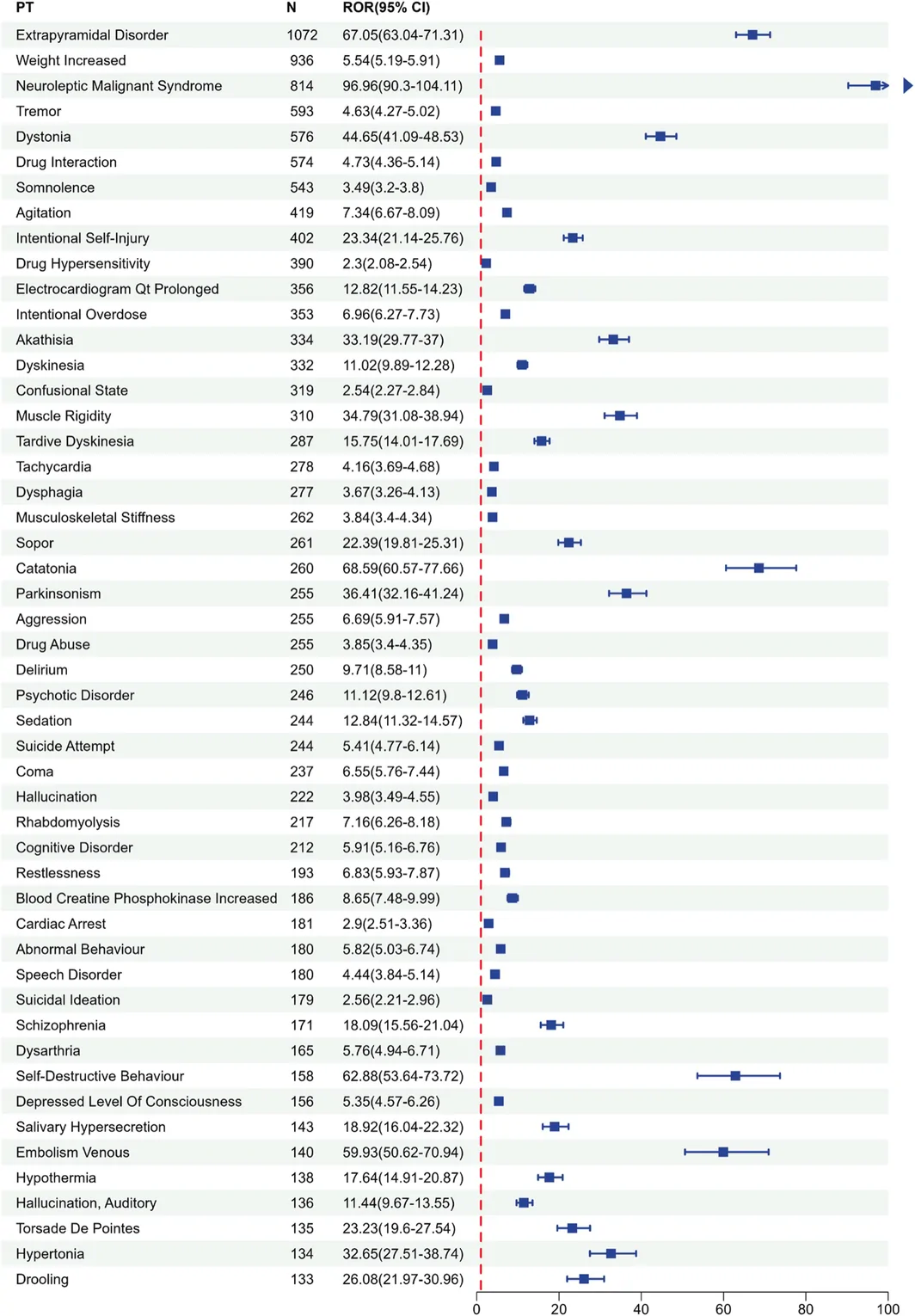
Visualizing the top 50 PTs by frequency.

**FIGURE 4:**
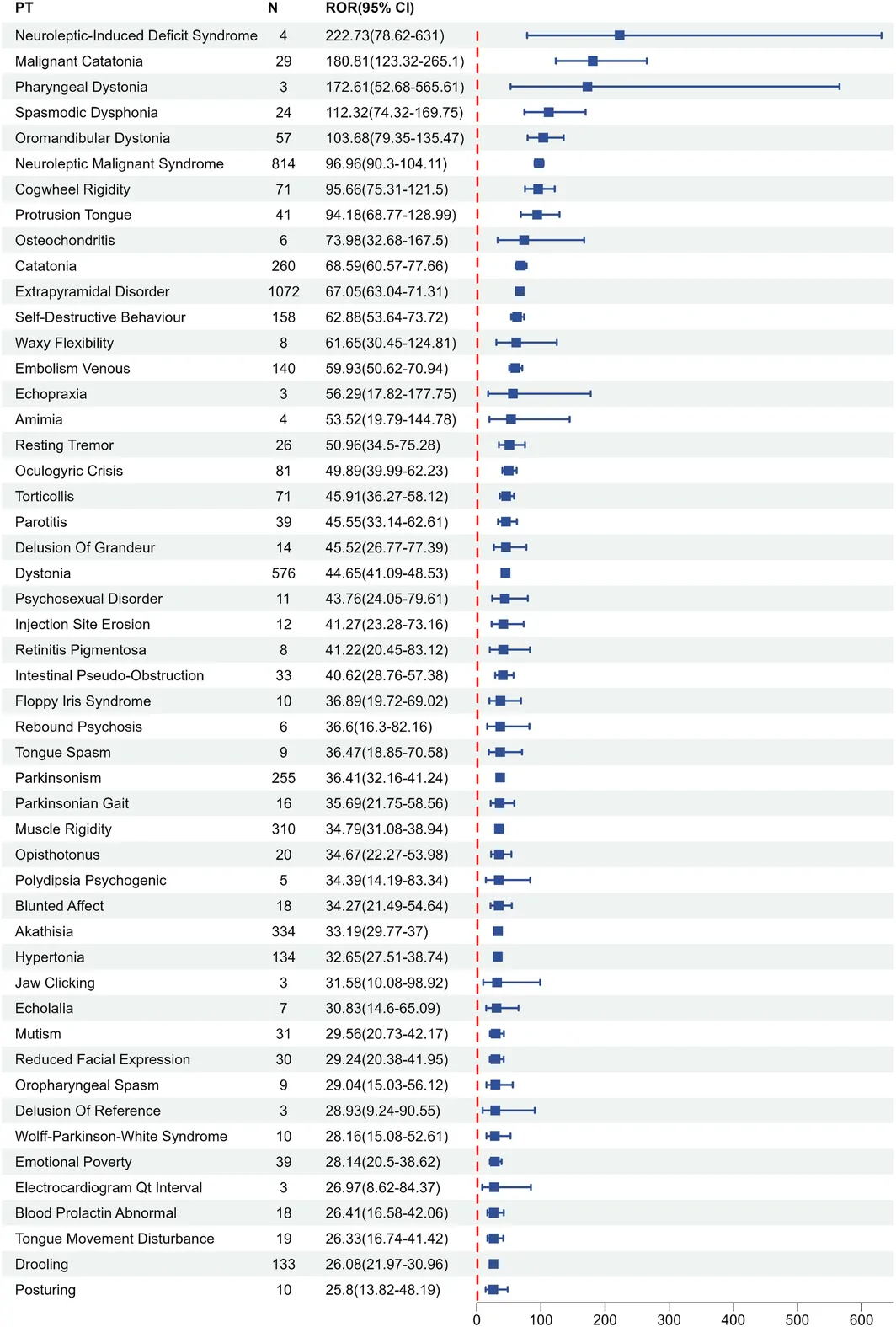
Visualizing the top 50 PTs by ROR value.

### 3.3 Subgroup Analysis

In the gender subgroup, as presented in Tables S3 and S4, male patients showed a higher reporting frequency for salivary hypersecretion, swollen tongue, drooling, pneumonia aspiration, delusion, and urinary retention. Whereas females were more likely to report confusional state, hypothermia, cardiac arrest, hyponatraemia, bradycardia, and respiratory failure. The age‐subgroup analysis indicated that dystonia was the predominant adverse event in pediatric patients. The most frequently reported adverse event in adult patients was increased weight. Drug interaction and confusional state were most prevalent in elderly patients. Detailed data are provided in Tables S5–S7.

### 3.4 Time to Onset and Weibull Distribution Analysis of Adverse Events

As illustrated in Figure 5, a total of 951 adverse events were included for analysis following the exclusion of reports with missing or erroneous start or onset dates. The majority of these events (*n* = 685, 72.03%) occurred within the initial 30 days following drug administration. The second most frequent interval was beyond 360 days (*n* = 102, 10.73%). The cumulative incidence of adverse events was depicted in Figure 6. The median time to onset for haloperidol‐related adverse events was 7 days, with an interquartile range of 40.5 days. Weibull distribution modeling indicated an “early failure” pattern, characterized by a decreasing hazard of event occurrence over time. However, the time‐to‐onset results should be interpreted with caution because of incomplete reporting details that may affect data representativeness and introduce bias. The corresponding analytical parameters were detailed in Table 4.

**FIGURE 5:**
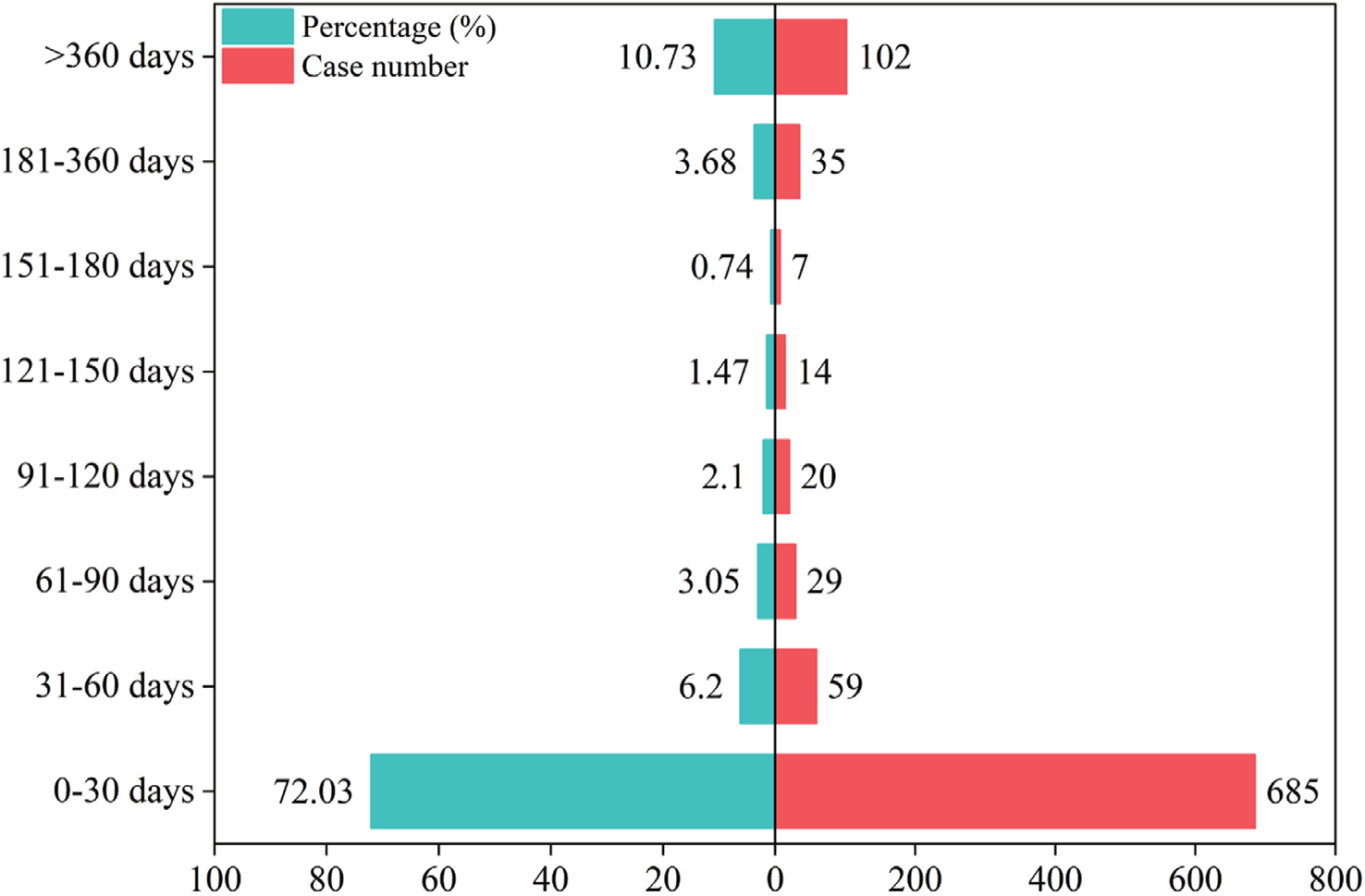
Time to onset of haloperidol‐related adverse events.

**FIGURE 6:**
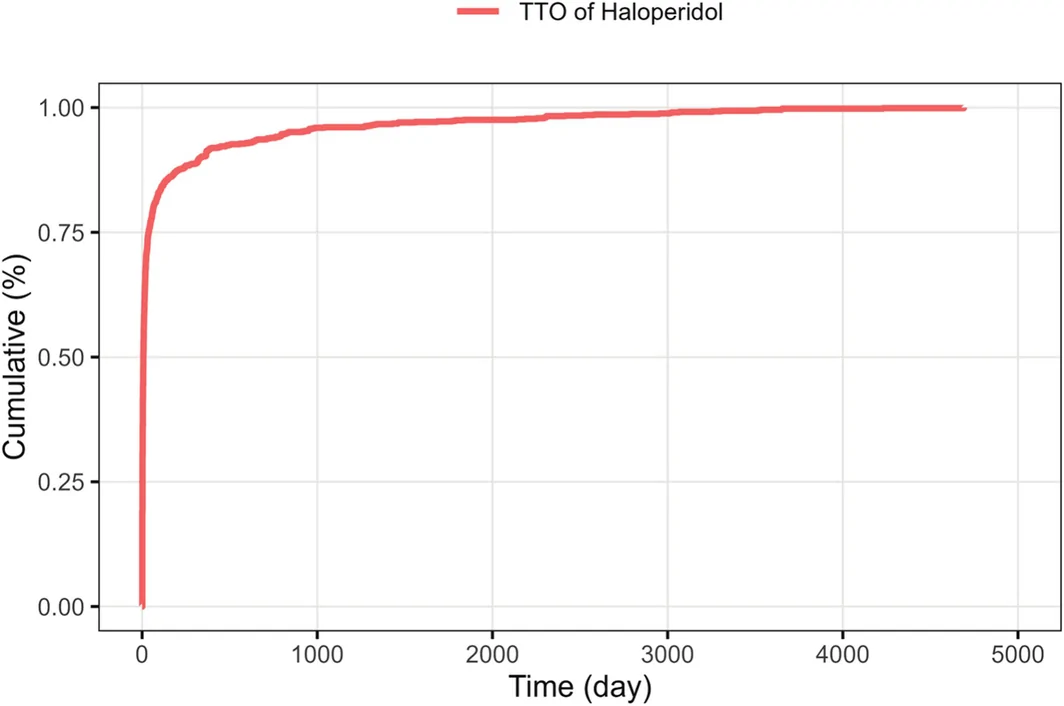
Cumulative incidence of haloperidol‐related adverse events over time. TTO, time to onset.

**TABLE 4:** Time to onset of haloperidol‐associated adverse events and Weibull distribution analysis.

Drug	TTO (days)		Weibull distribution		
	Case reports	Median (IQR)	Scale parameter: *α* (95% CI)	Shape parameter: *β* (95% CI)	Type
Haloperidol	951	7 (40.5)	40.43 (33.44, 47.43)	0.39 (0.37, 0.41)	Early failure

Abbreviations: CI, confidence interval; IQR, interquartile range; TTO, time to onset.

## 4 Discussion

### 4.1 Clinical Characteristics of Adverse Event Reports Associated With Haloperidol

In this study, male patients accounted for a higher proportion of adverse event reports than female patients, which is in line with earlier findings on sex differences in the prevalence of schizophrenia. Male rates of schizophrenia are around 40% greater than female rates, according to systematic studies and meta‐analyses [15, 16, 17]. The majority of reported patients were between 18 and 65 years of age, likely reflecting the higher incidence of schizophrenia within this age range [18, 19]. Geographically, reports were predominantly from the United States, which reflects the characteristics of FAERS as a U.S. drug adverse event reporting system and also suggests potential limitations regarding the racial representativeness of the data. In terms of clinical outcomes, a considerable proportion of reports involved serious outcomes, which points to a pronounced severity of certain adverse events and necessitates focused attention in clinical practice. Reports were mainly submitted by consumers and healthcare professionals, indicating active multi‐stakeholder participation. Temporally, the number of reports increased steadily between 2004 and 2015 before declining after 2016. Several factors could be responsible for this trend: as haloperidol's clinical use increased, its risk profile improved and its application became more standardized. At the same time, accumulated experience, the introduction of competing drugs, and potential reporting fatigue could all have contributed to the decline in the number of reports.

### 4.2 Signal Mining Analysis

Adverse events associated with haloperidol concentrated mainly in nervous and psychiatric disorders, which are closely related to its pharmacological effect of blocking the D2 receptor. Within the spectrum of neurological adverse events, extrapyramidal symptoms (EPS) have been consistently reported as the most frequent adverse events [20, 21]. In the current study, we identified a number of EPS‐related preferred terms, including extrapyramidal disorder, dystonia, tremor, akathisia, dyskinesia, parkinsonism, tardive dyskinesia, and hypertonia. According to meta‐analyses and clinical studies, 21% to 30% of haloperidol‐treated individuals had EPS [22, 23]. The primary pathogenic mechanism of haloperidol‐associated EPS stems from its antagonistic action on dopamine D2 receptors in the nigrostriatal pathway. By preventing dopamine from inhibiting acetylcholine release, this blockade causes excessive cholinergic activity in the basal ganglia, disrupting the dopaminergic–cholinergic balance and leading to EPS [24]. Notably, neuroleptic malignant syndrome (NMS) showed a remarkably strong signal intensity and high reporting frequency in our study. NMS is a potentially fatal condition characterized by muscle rigidity, altered consciousness, hyperthermia, and autonomic dysfunction [25, 26]. Furthermore, a number of adverse events that closely approximated the clinical characteristics of NMS were identified in our study, including muscle rigidity, catatonia, cogwheel rigidity, coma, depressed level of consciousness, and tachycardia. Moreover, prior research has demonstrated that the risk of haloperidol‐related EPS and NMS is greatly increased by large dosages or rapid dose escalation [27, 28, 29]. These findings suggest that when using haloperidol in clinical practice, the lowest effective dose should be employed with gradual titration. Clinicians should maintain surveillance for early symptoms such as fever, altered mental state, and muscular stiffness that could potentially indicate severe neurotoxicity. Early detection of these symptoms can help prevent more serious tolerability issues and enable intervention before treatment adherence is compromised or discontinuation is required.

Another system significantly affected by haloperidol was the psychiatric disorder category. Within this category, we detected signs that weren't specifically included on the package insert, including agitation, aggression, and delirium, as well as adverse effects that were in line with the package insert, like sedation, somnolence, restlessness, and abnormal behavior. Haloperidol's strong D2 receptor blockade in the mesolimbic and ascending reticular activating pathways is mainly responsible for its sedative effects [30]. Sedation may combine with other risk factors, such as serious illness, surgery, infection, or polypharmacy, in vulnerable people and might lead to delirium [31, 32]. Interpretation of agitation and aggression demanded considerable caution, as these manifestations could arise from the primary psychiatric condition or alternatively represent the clinical expression of drug‐associated akathisia. Akathisia, characterized by profound inner restlessness and subjective distress, could present as irritability, hostility, or even overtly aggressive behavior [33]. However, this link was frequently overlooked in routine clinical practice, leading to misdiagnosis as disease deterioration and consequent inappropriate dose escalation [33]. It was important to recognize that signal strength in the FAERS database reflected statistical associations between drugs and adverse events rather than causality. Therefore, although agitation and aggression showed strong signal linkages with haloperidol in this analysis, it could not be definitively determined whether these events were part of the underlying disease or its progression, or if they represented adverse reactions related to haloperidol. Nevertheless, given the potential of these adverse events to compromise treatment adherence and clinical outcomes, clinicians should maintain heightened vigilance for psychiatric‐related adverse reactions during haloperidol therapy, particularly those involving alterations in behavior and emotional state, to optimize patient management. Future studies are warranted to further elucidate the underlying mechanisms and contributing factors.

Additionally, several cardiovascular adverse events were identified in this study, such as electrocardiogram QT prolonged, tachycardia, cardiac arrest, and torsade de pointes, all of which showed relatively high reporting frequencies and signal strengths that warrant focused clinical attention. These findings are largely in accordance with prior studies documenting the cardiac effects of haloperidol, including reports of cardiac arrest associated with haloperidol [34]. According to studies by Nash et al. and Wang et al., between 9.9% and 14.2% of individuals receiving haloperidol experienced QT interval prolongation, with most cases progressing to severe prolongation [35, 36]. Experimental evidence suggests that these cardiac events may be related to haloperidol's effects on myocardial ion channels. In particular, haloperidol prolongs the action potential duration and the QTc interval by inhibiting the rapidly activating delayed rectifier potassium current (IKr/hERG) in cardiac myocytes [37, 38]. Additionally, the likelihood of developing torsades de pointes is significantly enhanced when paired with predisposing factors such as bradycardia, cardiac failure, hypokalemia, hypomagnesemia, or concurrent use of other QT‐prolonging drugs [39, 40, 41]. Based on these findings, we recommended enhanced electrocardiographic surveillance during haloperidol therapy. For patients with identifiable cardiovascular risk factors, comprising advanced age over 65, heart failure, coronary artery disease, or electrolyte disturbances containing hypokalemia, baseline electrocardiographic assessment before treatment initiation was advisable. When modifiable risk factors are present, clinicians should implement appropriate corrective measures before or during therapy. For high‐risk patients, if clinical conditions permit, other antipsychotic medications without QT‐prolonging effects may be a more sensible course of action.

Notably, our research identified many significant suicide‐related safety signals, such as intentional self‐injury, suicide attempt, suicidal ideation, and self‐destructive behavior, that are not included in the package insert. Although clinical studies have indicated haloperidol treatment does not substantially raise the risk of suicide death, it is still a challenge to establish a definitive association between haloperidol and suicidal ideation because of the short study periods and small sample sizes [42]. A cohort study showed that those who started using psychotropic drugs had more severe suicidal thoughts in the early stages of treatment than those who did not take these medications, but these thoughts tended to diminish over time with continued therapy [43]. Another pharmacoepidemiological study reported consistent results, showing a markedly higher risk of suicide and suicide attempt over the first 6 months after starting antipsychotics, especially in the first month [44]. However, a growing amount of research indicates that as antipsychotic treatment improves core psychotic symptoms, particularly in the cognitive and emotional domains, the overall risk of suicide eventually decreases with the longer course of treatment [45]. Additionally, suicidal behavior was influenced by multiple factors, such as incomplete symptom remission, undiagnosed bipolar disorder triggering hypomanic or manic states, disease progression, and comorbid depression [46]. Therefore, the interpretation of these signals required considerable caution. However, the notable frequency of suicide‐related adverse events observed in this study represented an important concern. Accordingly, clinicians should conduct comprehensive mental health assessments for patients receiving long‐term haloperidol therapy, with vigilant clinical monitoring and follow‐up to evaluate suicide risk, which frequently manifests during the initial phase of antipsychotic treatment.

Additional potentially novel adverse events were identified in this analysis, including weight increase, rhabdomyolysis, blood creatine phosphokinase increased, dysarthria, and embolism venous. Antipsychotic treatment has been associated with metabolic disturbances [47]. According to a meta‐analysis of 27 cohort studies, individuals treated with haloperidol experienced a weight increase of at least 7% above baseline [48]. The pharmacological processes that underlie weight increase associated with haloperidol are still not well understood. A possible explanation is that D2 receptor inhibition interferes with dopaminergic transmission in the mesolimbic reward pathway, potentially altering food reward perception and eating behavior, thereby contributing to weight increase [49]. Moreover, drug‐associated sedation and the subsequent decrease in physical activity may also contribute to this process. Thus, metabolic monitoring, such as regular weight assessment, should be taken into consideration during long‐term haloperidol administration. Rhabdomyolysis is a condition characterized by skeletal muscle cell degeneration and necrosis, leading to the release of intracellular contents into the bloodstream and urine, often accompanied by elevated blood creatine phosphokinase. Consistent with prior research, our results support the association between haloperidol and rhabdomyolysis [50, 51]. It has been suggested that muscle toxicity related to antipsychotics such as haloperidol may be mediated by serotonin and dopamine blockade [52]. Notably, this risk appeared to be dose‐dependent [53]. In the present study, embolism venous showed a relatively high frequency and signal strength. A meta‐analysis of 22 studies demonstrated that antipsychotic use significantly increased the risk of venous thromboembolism, particularly among obese patients, women, and individuals younger than 65 years [54]. Collectively, these findings offered new perspectives on haloperidol's safety profile and warranted attention and caution from healthcare professionals.

### 4.3 Subgroup Analysis of Safety Signals

Subgroup analysis revealed sex‐specific risks: female patients require vigilance for respiratory failure, hypothermia, hyponatremia, bradycardia, and cardiac arrest, while male patients need monitoring for urinary retention, aspiration pneumonia, hypersalivation, and drooling. Numerous interrelated factors may contribute to the observed sex variations in adverse event reporting. Previous research indicates that sex‐related variations in cardiac electrophysiology, pharmacokinetics, pharmacodynamics, and hormonal profiles may lead to different susceptibilities to various adverse reactions [55, 56, 57, 58]. Although the precise mechanisms underlying the sexual dimorphism in haloperidol‐related adverse effects are not fully elucidated, these variations should be carefully considered in therapeutic planning and monitoring. Age‐subgroup analysis revealed that dystonia predominated in pediatric patients, indicating a greater susceptibility to acute extrapyramidal symptoms and highlighting the necessity of careful dosage and attentive monitoring. Drug interactions and confusional state were the most frequently reported events in patients 65 years of age and older. This vulnerability was probably caused by the clinical complexity characteristic of this population, which included prevalent polypharmacy, age‐related decline in hepatic and renal function, and changes in blood–brain barrier function [59, 60]. As a result, using haloperidol in older individuals required more prudence, preferring lower starting dosages and thorough pharmacological assessment. These findings provided real‐world evidence for identifying high‐risk individuals and implementing targeted monitoring, while the exact biological mechanisms involved await further elucidation in prospective studies.

### 4.4 Time‐to‐Onset and Weibull Distribution Analysis of Adverse Events

The timing of adverse event onset was assessed using a Weibull distribution model. Results showed that most events (72.03%) occurred within the first month of haloperidol treatment, then declined. This aligned with the Weibull model's “early failure” pattern, indicating decreasing risk over time, underscoring the need for heightened vigilance early in therapy to improve patient safety. Interestingly, 14.41% of adverse events were recorded over 6 months after the start of treatment. These delayed‐onset events could be attributed to the late manifestation of toxic effects, the consequences of long‐term therapy, or individual variations leading to later reporting in some patients [61, 62]. This emphasized the necessity of long‐term monitoring to identify rare but clinically meaningful delayed adverse events. It should be noted that a large proportion of reports did not have full time‐to‐onset data, which might limit the accuracy of temporal pattern analysis. The resultant estimates still offer a reasonable approximation of the overall time‐to‐onset pattern since these missing data were probably caused by incomplete spontaneous reporting rather than systematic bias. However, while analyzing the time pattern of adverse reaction incidence, this restriction should be taken into account.

## 5 Limitations and Future Directions

There are a number of limitations to this study. First, since the FAERS database is a spontaneous reporting system, it is subject to reporting biases (e.g., awareness bias concerning adverse events), incomplete information (such as missing information on drug dose, duration of treatment, formulation, speed of administration, and concurrent drugs), and underreporting (such as the possibility that physicians and health care providers may not recognize certain symptoms as adverse events or fail to report them). This limitation could have affected the accuracy of the findings and impeded patient‐characteristic stratified assessments. In addition, subgroup analyses that highlight differences related to age and sex may be restricted by potential reporting biases and missing data and should be interpreted cautiously. Second, we are unable to establish a direct causal relationship or absolute risk between haloperidol use and the observed adverse events because the database data are observational. Pharmacovigilance signal identification primarily provides associative information and estimates the strength of a statistical association between haloperidol use and observed adverse events. Additional validation is necessary to confirm causation, such as extensive prospective cohort research, case–control analyses based on digital health records, and mechanism‐driven studies (cell experiments or animal models) to elucidate biological mechanisms. Additionally, we were unable to control for potential confounding factors, such as variations in baseline patient characteristics, because of the database's limitations. Third, the ability to perform thorough analyses of the timing of adverse events was severely limited by a low level of TTO reporting in FAERS (9.6%). This makes it necessary to interpret TTO results with caution. Fourth, a significant proportion of reports originated from the United States and Europe, with insufficient data representation from other regions. This might restrict the generalizability of findings to global populations and a variety of clinical contexts. Future studies should prioritize incorporating larger, more diverse datasets spanning multiple geographic regions to mitigate potential biases and enhance representativeness. Concurrently, rigorously designed prospective studies combining data from clinical trials with real‐world epidemiological evidence are warranted to provide a more comprehensive safety assessment of haloperidol.

## 6 Conclusion

In conclusion, this study provides comprehensive real‐world post‐marketing safety data on haloperidol, serving as a valuable complement to clinical trial information. We not only confirmed known neuropsychiatric and cardiac adverse events associated with haloperidol but also identified several potential new safety signals. These findings underscore the need for heightened clinical vigilance, particularly during the first month of treatment, to facilitate early intervention and improve patient outcomes. Furthermore, the study highlights the importance of individualized, risk‐stratified monitoring. Prospective studies are warranted to validate these findings and further clarify the safety profile of haloperidol.

## Author Contributions

Xiang Liu, Ying Liu, Nengping Luo, and Lu Xiao designed the research. Xiang Liu and Ying Liu collected and analyzed the data and drafted the manuscript. Nengping Luo, Lu Xiao, and Xiang Liu revised the manuscript. All authors contributed to the article and approved the submitted version.

## Funding

The authors have nothing to report.

## Ethics Statement

Ethical approval was not required, as all data were publicly accessible and fully de‐identified.

## Consent

All of the authors are aware of and agree to the content of the paper and their being listed as co‐authors of the paper.

## Conflicts of Interest

The authors declare no conflicts of interest.

## Supporting information


**Table S1:** Fourfold table of disproportionality analysis.
**Table S2:** Formula and criteria of four algorithms for adverse event signal detection.
**Table S3:** Top 50 most frequent adverse events for haloperidol at the preferred term (PT) level in males from FAERS data.
**Table S4:** Top 50 most frequent adverse events for haloperidol at the preferred term (PT) level in females from FAERS data.
**Table S5:** Adverse events at the preferred term (PT) level for haloperidol in patients aged under 18 from FAERS data.
**Table S6:** Adverse events at the preferred term (PT) level for haloperidol in patients aged 18 to 65 from FAERS data.
**Table S7:** Adverse events at the preferred term (PT) level for haloperidol in patients aged over 65 from FAERS data.

## Data Availability

The data that support the findings of this study are openly available in the FDA Adverse Event Reporting System at https://www.fda.gov.
