# Supplementary material for: The Real‐World Safety Profile of Haloperidol: A Comprehensive Pharmacovigilance Analysis Using the Large‐Scale FAERS Database
Source: CNS Neurosci Ther. 2026 Oct 20;32(10):e71217. doi: 10.1002/cns.71217 (PMC13682282; doi:10.1002/cns.71217)
Supplement: Supplementary file 1 [file CNS-32-e71217-s001.docx]

**Supplementary Table 1**. Fourfold table of disproportionality analysis.

|  | Reports with target adverse event | Reports with other adverse events |
| --- | --- | --- |
| Reports as the primary suspected drug | a | b |
| Reports with other drugs as the primary suspected drug | c | d |

Abbreviation: a, number of reports containing both target drug and target adverse event; b, number of reports containing other adverse events of target drug; c, number of reports containing target adverse event of other drugs; d, number of reports containing other drugs and other adverse events

**Supplementary Table 2**. Formula and criteria of four algorithms for adverse event signal detection.

| Algorithm | Formula | Criteria |
| --- | --- | --- |
| ROR | ROR = $\frac{a/c}{b/d}$  95% CI = $e^{\ln(ROR)\pm1.96\sqrt{\frac{1}{a}+\frac{1}{b}+\frac{1}{c}+\frac{1}{d}}}$ | N ≥ 3,  Lower limit of 95% CI > 1 |
| PRR | PRR = $\frac{a}{a+b}/\frac{c}{c+d}$  χ^2^ = $\frac{[(ad-bc)^2](a+b+c+d)}{[(a+b)(c+d)(a+c)(b+d)}$ | PRR≥2, χ^2^≥4, N≥3 |
| BCPNN | IC = log_2_$\frac{a(a+b+c+d)}{(a+c)(a+b)}$  IC025 = $e^{\ln(IC)-1.96\sqrt{\frac{1}{a}+\frac{1}{b}+\frac{1}{c}+\frac{1}{d}}}$ | IC025 > 0 |
| MGPS | EBGM = $\frac{a(a+b+c+d)}{(a+c)(a+b)}$  EBGM05 = $e^{\ln(EBGM)-1.96\sqrt{\frac{1}{a}+\frac{1}{b}+\frac{1}{c}+\frac{1}{d}}}$ | EBGM05>2 |

Abbreviation: N, number of reports; ROR, reporting odds ratio; CI, confidence interval; PRR, proportional reporting ratio; χ2, chi-squared; IC, information component; IC025, the lower limit of 95%CI of the IC; EBGM, empirical bayesian geometric mean; EBGM05, lower limit of 95% confidence interval of EBGM.

**Supplementary Table 3**. Top 50 most frequent adverse events for haloperidol at the preferred term (PT) level in males from FAERS data.

| PT | Case numbers | ROR(95%CI) | PRR(χ^2^) | EBGM(EBGM05) | IC(IC025) |
| --- | --- | --- | --- | --- | --- |
| Extrapyramidal Disorder | 602 | 63.46 ( 58.4 - 68.95 ) | 61.89 ( 34238.73 ) | 58.78 ( 54.1 ) | 5.88 ( 5.62 ) |
| Neuroleptic Malignant Syndrome | 474 | 69.13 ( 62.95 - 75.9 ) | 67.78 ( 29461.77 ) | 64.07 ( 58.35 ) | 6 ( 5.68 ) |
| Weight Increased | 451 | 6.28 ( 5.72 - 6.9 ) | 6.18 ( 1954.28 ) | 6.15 ( 5.6 ) | 2.62 ( 2.47 ) |
| Dystonia | 342 | 48.07 ( 43.11 - 53.6 ) | 47.4 ( 14925.19 ) | 45.57 ( 40.87 ) | 5.51 ( 5.17 ) |
| Tremor | 301 | 4.94 ( 4.41 - 5.54 ) | 4.89 ( 930.33 ) | 4.88 ( 4.35 ) | 2.29 ( 2.1 ) |
| Somnolence | 257 | 3.23 ( 2.86 - 3.66 ) | 3.21 ( 390.87 ) | 3.2 ( 2.83 ) | 1.68 ( 1.49 ) |
| Drug Interaction | 256 | 3.33 ( 2.94 - 3.76 ) | 3.3 ( 411.16 ) | 3.3 ( 2.91 ) | 1.72 ( 1.53 ) |
| Agitation | 233 | 6.44 ( 5.65 - 7.32 ) | 6.38 ( 1053.52 ) | 6.35 ( 5.58 ) | 2.67 ( 2.44 ) |
| Dyskinesia | 186 | 11.03 ( 9.54 - 12.75 ) | 10.95 ( 1667.65 ) | 10.86 ( 9.39 ) | 3.44 ( 3.15 ) |
| Intentional Self-Injury | 175 | 21.03 ( 18.1 - 24.43 ) | 20.88 ( 3254.8 ) | 20.53 ( 17.67 ) | 4.36 ( 3.99 ) |
| Muscle Rigidity | 173 | 29.44 ( 25.3 - 34.25 ) | 29.24 ( 4601.97 ) | 28.54 ( 24.53 ) | 4.83 ( 4.4 ) |
| Dysphagia | 168 | 4.06 ( 3.48 - 4.72 ) | 4.03 ( 382.67 ) | 4.02 ( 3.46 ) | 2.01 ( 1.76 ) |
| Tachycardia | 162 | 4.58 ( 3.92 - 5.34 ) | 4.55 ( 448.22 ) | 4.54 ( 3.89 ) | 2.18 ( 1.92 ) |
| Intentional Overdose | 161 | 6.42 ( 5.5 - 7.5 ) | 6.39 ( 728.21 ) | 6.36 ( 5.44 ) | 2.67 ( 2.39 ) |
| Aggression | 160 | 5.1 ( 4.37 - 5.96 ) | 5.08 ( 522.23 ) | 5.06 ( 4.33 ) | 2.34 ( 2.07 ) |
| Akathisia | 152 | 27.35 ( 23.27 - 32.14 ) | 27.18 ( 3745.47 ) | 26.58 ( 22.62 ) | 4.73 ( 4.27 ) |
| Drug Hypersensitivity | 148 | 2.8 ( 2.38 - 3.29 ) | 2.78 ( 169.24 ) | 2.78 ( 2.36 ) | 1.48 ( 1.22 ) |
| Tardive Dyskinesia | 148 | 17.94 ( 15.25 - 21.12 ) | 17.84 ( 2317.45 ) | 17.58 ( 14.94 ) | 4.14 ( 3.74 ) |
| Psychotic Disorder | 138 | 9.19 ( 7.77 - 10.87 ) | 9.14 ( 993.22 ) | 9.08 ( 7.67 ) | 3.18 ( 2.85 ) |
| Rhabdomyolysis | 137 | 5.5 ( 4.64 - 6.5 ) | 5.47 ( 498.66 ) | 5.45 ( 4.61 ) | 2.45 ( 2.15 ) |
| Electrocardiogram Qt Prolonged | 136 | 9.42 ( 7.95 - 11.15 ) | 9.37 ( 1009.17 ) | 9.3 ( 7.85 ) | 3.22 ( 2.88 ) |
| Suicide Attempt | 135 | 5.67 ( 4.79 - 6.72 ) | 5.65 ( 514.18 ) | 5.62 ( 4.75 ) | 2.49 ( 2.19 ) |
| Parkinsonism | 132 | 30.36 ( 25.53 - 36.11 ) | 30.2 ( 3632.3 ) | 29.45 ( 24.77 ) | 4.88 ( 4.35 ) |
| Catatonia | 130 | 49.43 ( 41.45 - 58.94 ) | 49.17 ( 5883.8 ) | 47.19 ( 39.58 ) | 5.56 ( 4.87 ) |
| Sedation | 128 | 11.14 ( 9.36 - 13.27 ) | 11.09 ( 1164.43 ) | 10.99 ( 9.23 ) | 3.46 ( 3.09 ) |
| Drug Abuse | 126 | 2.52 ( 2.11 - 3 ) | 2.51 ( 114.17 ) | 2.5 ( 2.1 ) | 1.32 ( 1.05 ) |
| Delirium | 122 | 6.83 ( 5.72 - 8.17 ) | 6.8 ( 600.82 ) | 6.77 ( 5.66 ) | 2.76 ( 2.43 ) |
| Musculoskeletal Stiffness | 121 | 4.17 ( 3.49 - 4.99 ) | 4.16 ( 289.55 ) | 4.15 ( 3.47 ) | 2.05 ( 1.75 ) |
| Blood Creatine Phosphokinase Increased | 116 | 6.39 ( 5.32 - 7.67 ) | 6.36 ( 521.42 ) | 6.33 ( 5.27 ) | 2.66 ( 2.33 ) |
| Schizophrenia | 112 | 14.7 ( 12.2 - 17.72 ) | 14.64 ( 1405.56 ) | 14.47 ( 12 ) | 3.85 ( 3.42 ) |
| Sopor | 110 | 19.45 ( 16.11 - 23.5 ) | 19.37 ( 1885.01 ) | 19.06 ( 15.78 ) | 4.25 ( 3.76 ) |
| Cognitive Disorder | 109 | 5.49 ( 4.55 - 6.63 ) | 5.47 ( 396.76 ) | 5.45 ( 4.51 ) | 2.45 ( 2.11 ) |
| Coma | 107 | 4.84 ( 4 - 5.86 ) | 4.83 ( 323.4 ) | 4.81 ( 3.98 ) | 2.27 ( 1.94 ) |
| Hallucination | 106 | 2.83 ( 2.34 - 3.43 ) | 2.82 ( 124.58 ) | 2.82 ( 2.33 ) | 1.49 ( 1.19 ) |
| Speech Disorder | 104 | 4.76 ( 3.93 - 5.78 ) | 4.75 ( 306.7 ) | 4.73 ( 3.9 ) | 2.24 ( 1.91 ) |
| Abnormal Behaviour | 98 | 4.21 ( 3.45 - 5.14 ) | 4.2 ( 238.26 ) | 4.19 ( 3.43 ) | 2.07 ( 1.73 ) |
| Restlessness | 93 | 5.33 ( 4.35 - 6.54 ) | 5.32 ( 324.54 ) | 5.3 ( 4.32 ) | 2.4 ( 2.04 ) |
| Dysarthria | 87 | 5.08 ( 4.11 - 6.27 ) | 5.06 ( 282.77 ) | 5.05 ( 4.09 ) | 2.34 ( 1.96 ) |
| Salivary Hypersecretion | 87 | 17.33 ( 14.02 - 21.43 ) | 17.27 ( 1314.38 ) | 17.03 ( 13.78 ) | 4.09 ( 3.54 ) |
| Altered State Of Consciousness | 81 | 7.74 ( 6.22 - 9.64 ) | 7.72 ( 470.91 ) | 7.68 ( 6.17 ) | 2.94 ( 2.51 ) |
| Swollen Tongue | 79 | 8.27 ( 6.63 - 10.32 ) | 8.25 ( 499.7 ) | 8.2 ( 6.57 ) | 3.03 ( 2.59 ) |
| Drooling | 76 | 23.42 ( 18.66 - 29.41 ) | 23.35 ( 1594.01 ) | 22.91 ( 18.25 ) | 4.52 ( 3.82 ) |
| Hypertonia | 76 | 26.38 ( 21.01 - 33.12 ) | 26.3 ( 1808.39 ) | 25.73 ( 20.49 ) | 4.69 ( 3.95 ) |
| Pneumonia Aspiration | 74 | 4.72 ( 3.75 - 5.93 ) | 4.7 ( 215.1 ) | 4.69 ( 3.73 ) | 2.23 ( 1.83 ) |
| Hallucination, Auditory | 73 | 9.6 ( 7.62 - 12.09 ) | 9.57 ( 555.91 ) | 9.5 ( 7.54 ) | 3.25 ( 2.75 ) |
| Depressed Level Of Consciousness | 73 | 4.08 ( 3.24 - 5.14 ) | 4.07 ( 168.59 ) | 4.06 ( 3.22 ) | 2.02 ( 1.63 ) |
| Unresponsive To Stimuli | 63 | 4.87 ( 3.81 - 6.25 ) | 4.86 ( 192.72 ) | 4.85 ( 3.78 ) | 2.28 ( 1.83 ) |
| Delusion | 59 | 6.89 ( 5.34 - 8.91 ) | 6.88 ( 294.86 ) | 6.85 ( 5.3 ) | 2.78 ( 2.27 ) |
| Self-Destructive Behaviour | 59 | 36.97 ( 28.52 - 47.92 ) | 36.88 ( 1995.71 ) | 35.77 ( 27.59 ) | 5.16 ( 4.12 ) |
| Urinary Retention | 58 | 2.9 ( 2.24 - 3.75 ) | 2.89 ( 71.79 ) | 2.89 ( 2.23 ) | 1.53 ( 1.11 ) |

Abbreviation: ROR, reporting odds ratio; PRR, proportional reporting ratio; EBGM, empirical Bayesian geometric mean; EBGM05, the lower limit of the 95% CI of EBGM; IC, information component; IC025, the lower limit of the 95% CI of the IC; CI, confidence interval; PT,preferred term.

**Supplementary Table 4**. Top 50 most frequent adverse events for haloperidol at the preferred term (PT) level in females from FAERS data.

| PT | Case numbers | ROR(95%CI) | PRR(χ2) | EBGM(EBGM05) | IC(IC025) |
| --- | --- | --- | --- | --- | --- |
| Weight Increased | 442 | 6.09 ( 5.54 - 6.69 ) | 5.97 ( 1831.8 ) | 5.96 ( 5.42 ) | 2.57 ( 2.42 ) |
| Extrapyramidal Disorder | 383 | 67.73 ( 61.13 - 75.04 ) | 66.36 ( 24025.09 ) | 64.67 ( 58.37 ) | 6.01 ( 5.64 ) |
| Neuroleptic Malignant Syndrome | 278 | 135.38 ( 119.89 - 152.88 ) | 133.38 ( 34678.49 ) | 126.67 ( 112.18 ) | 6.98 ( 6.27 ) |
| Tremor | 241 | 4.47 ( 3.94 - 5.08 ) | 4.43 ( 640.72 ) | 4.42 ( 3.9 ) | 2.15 ( 1.94 ) |
| Intentional Self-Injury | 218 | 30.17 ( 26.38 - 34.51 ) | 29.83 ( 6005.23 ) | 29.49 ( 25.78 ) | 4.88 ( 4.51 ) |
| Drug Interaction | 216 | 5.57 ( 4.87 - 6.37 ) | 5.52 ( 798.37 ) | 5.51 ( 4.81 ) | 2.46 ( 2.23 ) |
| Somnolence | 211 | 3.52 ( 3.07 - 4.03 ) | 3.49 ( 374.91 ) | 3.48 ( 3.04 ) | 1.8 ( 1.58 ) |
| Intentional Overdose | 184 | 9.53 ( 8.24 - 11.03 ) | 9.45 ( 1386.28 ) | 9.42 ( 8.14 ) | 3.24 ( 2.96 ) |
| Dystonia | 165 | 34.09 ( 29.21 - 39.78 ) | 33.8 ( 5182.48 ) | 33.36 ( 28.59 ) | 5.06 ( 4.58 ) |
| Agitation | 159 | 8.33 ( 7.13 - 9.74 ) | 8.27 ( 1013.64 ) | 8.24 ( 7.05 ) | 3.04 ( 2.75 ) |
| Sopor | 149 | 30.28 ( 25.75 - 35.61 ) | 30.05 ( 4135.47 ) | 29.7 ( 25.26 ) | 4.89 ( 4.4 ) |
| Confusional State | 148 | 3.18 ( 2.71 - 3.74 ) | 3.17 ( 219.73 ) | 3.16 ( 2.69 ) | 1.66 ( 1.4 ) |
| Electrocardiogram Qt Prolonged | 135 | 13.72 ( 11.58 - 16.26 ) | 13.63 ( 1572.36 ) | 13.56 ( 11.44 ) | 3.76 ( 3.38 ) |
| Musculoskeletal Stiffness | 131 | 4.27 ( 3.6 - 5.07 ) | 4.25 ( 325.19 ) | 4.24 ( 3.57 ) | 2.08 ( 1.8 ) |
| Coma | 120 | 9.35 ( 7.81 - 11.19 ) | 9.29 ( 885.36 ) | 9.26 ( 7.74 ) | 3.21 ( 2.85 ) |
| Dyskinesia | 120 | 10.56 ( 8.82 - 12.64 ) | 10.5 ( 1027.41 ) | 10.46 ( 8.74 ) | 3.39 ( 3.01 ) |
| Akathisia | 119 | 36.18 ( 30.18 - 43.39 ) | 35.96 ( 3987.93 ) | 35.46 ( 29.58 ) | 5.15 ( 4.52 ) |
| Muscle Rigidity | 119 | 39.63 ( 33.05 - 47.52 ) | 39.38 ( 4383.28 ) | 38.79 ( 32.34 ) | 5.28 ( 4.62 ) |
| Drug Abuse | 118 | 6.62 ( 5.53 - 7.94 ) | 6.59 ( 558.39 ) | 6.57 ( 5.48 ) | 2.72 ( 2.38 ) |
| Catatonia | 115 | 100.22 ( 83.14 - 120.83 ) | 99.61 ( 10796.65 ) | 95.83 ( 79.49 ) | 6.58 ( 5.45 ) |
| Tachycardia | 108 | 4.18 ( 3.46 - 5.05 ) | 4.16 ( 259.08 ) | 4.15 ( 3.44 ) | 2.05 ( 1.74 ) |
| Tardive Dyskinesia | 106 | 15.22 ( 12.57 - 18.44 ) | 15.14 ( 1392.12 ) | 15.06 ( 12.43 ) | 3.91 ( 3.45 ) |
| Dysphagia | 99 | 3.51 ( 2.88 - 4.28 ) | 3.5 ( 176.85 ) | 3.5 ( 2.87 ) | 1.81 ( 1.48 ) |
| Self-Destructive Behaviour | 97 | 107.7 ( 87.85 - 132.04 ) | 107.15 ( 9780.86 ) | 102.78 ( 83.83 ) | 6.68 ( 5.36 ) |
| Hallucination | 94 | 5.16 ( 4.21 - 6.31 ) | 5.13 ( 312.59 ) | 5.13 ( 4.18 ) | 2.36 ( 2 ) |
| Suicide Attempt | 92 | 5.37 ( 4.37 - 6.59 ) | 5.34 ( 324.51 ) | 5.34 ( 4.35 ) | 2.42 ( 2.05 ) |
| Delirium | 88 | 11.64 ( 9.43 - 14.36 ) | 11.59 ( 847.74 ) | 11.54 ( 9.35 ) | 3.53 ( 3.06 ) |
| Psychotic Disorder | 86 | 12.67 ( 10.25 - 15.67 ) | 12.62 ( 915.78 ) | 12.56 ( 10.16 ) | 3.65 ( 3.16 ) |
| Cognitive Disorder | 84 | 6.05 ( 4.88 - 7.5 ) | 6.03 ( 351.83 ) | 6.02 ( 4.86 ) | 2.59 ( 2.19 ) |
| Parkinsonism | 83 | 37.94 ( 30.53 - 47.15 ) | 37.78 ( 2927.67 ) | 37.23 ( 29.96 ) | 5.22 ( 4.38 ) |
| Aggression | 79 | 8.08 ( 6.48 - 10.08 ) | 8.05 ( 486.65 ) | 8.03 ( 6.44 ) | 3.01 ( 2.56 ) |
| Hypothermia | 77 | 28.72 ( 22.93 - 35.97 ) | 28.61 ( 2028.63 ) | 28.3 ( 22.59 ) | 4.82 ( 4.06 ) |
| Restlessness | 75 | 7.48 ( 5.96 - 9.39 ) | 7.45 ( 418.11 ) | 7.44 ( 5.92 ) | 2.89 ( 2.44 ) |
| Rhabdomyolysis | 73 | 10.07 ( 8 - 12.68 ) | 10.04 ( 591.85 ) | 10 ( 7.94 ) | 3.32 ( 2.82 ) |
| Depressed Level Of Consciousness | 72 | 6.89 ( 5.47 - 8.69 ) | 6.87 ( 360.41 ) | 6.85 ( 5.44 ) | 2.78 ( 2.33 ) |
| Speech Disorder | 71 | 4.32 ( 3.42 - 5.46 ) | 4.31 ( 180.33 ) | 4.3 ( 3.41 ) | 2.11 ( 1.7 ) |
| Dysarthria | 69 | 6.58 ( 5.19 - 8.33 ) | 6.56 ( 324.22 ) | 6.54 ( 5.16 ) | 2.71 ( 2.25 ) |
| Suicidal Ideation | 69 | 2.6 ( 2.06 - 3.3 ) | 2.6 ( 67.86 ) | 2.6 ( 2.05 ) | 1.38 ( 1 ) |
| Cardiac Arrest | 66 | 3.49 ( 2.74 - 4.45 ) | 3.48 ( 116.8 ) | 3.48 ( 2.73 ) | 1.8 ( 1.39 ) |
| Hyponatraemia | 66 | 4.15 ( 3.26 - 5.28 ) | 4.14 ( 156.91 ) | 4.13 ( 3.24 ) | 2.05 ( 1.63 ) |
| Abnormal Behaviour | 66 | 7.42 ( 5.82 - 9.45 ) | 7.4 ( 364.18 ) | 7.38 ( 5.79 ) | 2.88 ( 2.4 ) |
| Sedation | 65 | 10.11 ( 7.92 - 12.91 ) | 10.08 ( 529.85 ) | 10.05 ( 7.87 ) | 3.33 ( 2.79 ) |
| Torsade De Pointes | 64 | 28.14 ( 21.98 - 36.01 ) | 28.04 ( 1650.8 ) | 27.74 ( 21.68 ) | 4.79 ( 3.94 ) |
| Blood Creatine Phosphokinase Increased | 62 | 12.12 ( 9.44 - 15.57 ) | 12.09 ( 627.62 ) | 12.03 ( 9.37 ) | 3.59 ( 2.99 ) |
| Hallucination, Auditory | 55 | 13.48 ( 10.34 - 17.58 ) | 13.45 ( 630.39 ) | 13.38 ( 10.26 ) | 3.74 ( 3.07 ) |
| Hypertonia | 53 | 42.9 ( 32.68 - 56.3 ) | 42.78 ( 2126.14 ) | 42.07 ( 32.06 ) | 5.39 ( 4.18 ) |
| Bradycardia | 49 | 3.84 ( 2.9 - 5.09 ) | 3.84 ( 102.63 ) | 3.83 ( 2.89 ) | 1.94 ( 1.45 ) |
| Schizophrenia | 49 | 20.27 ( 15.29 - 26.85 ) | 20.22 ( 887.91 ) | 20.06 ( 15.14 ) | 4.33 ( 3.45 ) |
| Embolism Venous | 49 | 70.81 ( 53.29 - 94.09 ) | 70.63 ( 3270.95 ) | 68.71 ( 51.71 ) | 6.1 ( 4.45 ) |

Abbreviation: ROR, reporting odds ratio; PRR, proportional reporting ratio; EBGM, empirical Bayesian geometric mean; EBGM05, the lower limit of the 95% CI of EBGM; IC, information component; IC025, the lower limit of the 95% CI of the IC; CI, confidence interval; PT, preferred term.

**Supplementary Table 5**. Adverse events at the PT level for haloperidol in patients aged under 18 from FAERS data.

| PT | Case numbers | ROR(95%CI) | PRR(χ2) | EBGM(EBGM05) | IC(IC025) |
| --- | --- | --- | --- | --- | --- |
| Dystonia | 95 | 42.33 ( 34.37 - 52.13 ) | 40.95 ( 3571.07 ) | 39.5 ( 32.07 ) | 5.3 ( 4.51 ) |
| Extrapyramidal Disorder | 73 | 47.24 ( 37.27 - 59.89 ) | 46.06 ( 3088.58 ) | 44.22 ( 34.89 ) | 5.47 ( 4.46 ) |
| Tremor | 61 | 9.45 ( 7.32 - 12.19 ) | 9.27 ( 447.2 ) | 9.2 ( 7.13 ) | 3.2 ( 2.65 ) |
| Somnolence | 49 | 3.43 ( 2.59 - 4.55 ) | 3.39 ( 82.75 ) | 3.38 ( 2.55 ) | 1.76 ( 1.28 ) |
| Neuroleptic Malignant Syndrome | 44 | 54.02 ( 39.82 - 73.28 ) | 53.2 ( 2148.97 ) | 50.76 ( 37.42 ) | 5.67 ( 4.15 ) |
| Agitation | 37 | 4.73 ( 3.41 - 6.54 ) | 4.68 ( 106.8 ) | 4.66 ( 3.37 ) | 2.22 ( 1.62 ) |
| Catatonia | 36 | 65.13 ( 46.44 - 91.35 ) | 64.32 ( 2119.05 ) | 60.78 ( 43.34 ) | 5.93 ( 4.05 ) |
| Oculogyric Crisis | 36 | 68.88 ( 49.09 - 96.65 ) | 68.02 ( 2237.5 ) | 64.07 ( 45.66 ) | 6 ( 4.07 ) |
| Dyskinesia | 33 | 10.57 ( 7.49 - 14.92 ) | 10.46 ( 279.93 ) | 10.37 ( 7.34 ) | 3.37 ( 2.52 ) |
| Altered State Of Consciousness | 32 | 19.66 ( 13.84 - 27.95 ) | 19.45 ( 550.64 ) | 19.13 ( 13.46 ) | 4.26 ( 3.12 ) |
| Dysphagia | 28 | 8.26 ( 5.68 - 12 ) | 8.19 ( 175.51 ) | 8.13 ( 5.6 ) | 3.02 ( 2.17 ) |
| Drug Interaction | 27 | 3.53 ( 2.41 - 5.15 ) | 3.5 ( 48.23 ) | 3.49 ( 2.39 ) | 1.8 ( 1.13 ) |
| Salivary Hypersecretion | 27 | 33.62 ( 22.89 - 49.4 ) | 33.32 ( 821.34 ) | 32.35 ( 22.02 ) | 5.02 ( 3.37 ) |
| Hyperreflexia | 25 | 45.73 ( 30.6 - 68.35 ) | 45.34 ( 1040.82 ) | 43.56 ( 29.15 ) | 5.45 ( 3.47 ) |
| Restlessness | 24 | 9.13 ( 6.1 - 13.67 ) | 9.06 ( 170.9 ) | 9 ( 6.01 ) | 3.17 ( 2.19 ) |
| Intentional Self-Injury | 23 | 5.69 ( 3.77 - 8.59 ) | 5.65 ( 87.75 ) | 5.63 ( 3.73 ) | 2.49 ( 1.64 ) |
| Muscle Rigidity | 22 | 25.22 ( 16.5 - 38.56 ) | 25.04 ( 496.41 ) | 24.5 ( 16.03 ) | 4.61 ( 2.99 ) |
| Hyperhidrosis | 22 | 6.84 ( 4.49 - 10.41 ) | 6.79 ( 108.11 ) | 6.76 ( 4.44 ) | 2.76 ( 1.83 ) |
| Drooling | 21 | 26.83 ( 17.37 - 41.43 ) | 26.64 ( 505.93 ) | 26.02 ( 16.85 ) | 4.7 ( 2.98 ) |
| Hypertonia | 21 | 19.89 ( 12.9 - 30.68 ) | 19.75 ( 367.35 ) | 19.42 ( 12.59 ) | 4.28 ( 2.78 ) |
| Sedation | 20 | 13.11 ( 8.42 - 20.41 ) | 13.03 ( 219.58 ) | 12.89 ( 8.28 ) | 3.69 ( 2.4 ) |
| Stupor | 20 | 83.69 ( 53.03 - 132.07 ) | 83.11 ( 1507.11 ) | 77.27 ( 48.96 ) | 6.27 ( 3.4 ) |
| Gait Disturbance | 19 | 4.66 ( 2.96 - 7.32 ) | 4.63 ( 54.01 ) | 4.62 ( 2.94 ) | 2.21 ( 1.32 ) |
| Dysarthria | 19 | 10.64 ( 6.76 - 16.75 ) | 10.58 ( 163.3 ) | 10.49 ( 6.66 ) | 3.39 ( 2.18 ) |
| Musculoskeletal Stiffness | 18 | 11.12 ( 6.98 - 17.72 ) | 11.06 ( 163.16 ) | 10.96 ( 6.88 ) | 3.45 ( 2.18 ) |
| Akathisia | 16 | 21.59 ( 13.15 - 35.46 ) | 21.48 ( 306.39 ) | 21.08 ( 12.83 ) | 4.4 ( 2.56 ) |
| Depressed Level Of Consciousness | 16 | 4.71 ( 2.88 - 7.71 ) | 4.69 ( 46.35 ) | 4.68 ( 2.86 ) | 2.23 ( 1.24 ) |
| Electrocardiogram Qt Prolonged | 16 | 5.44 ( 3.33 - 8.91 ) | 5.42 ( 57.44 ) | 5.4 ( 3.3 ) | 2.43 ( 1.4 ) |
| Torticollis | 15 | 40.88 ( 24.38 - 68.54 ) | 40.67 ( 559.55 ) | 39.24 ( 23.4 ) | 5.29 ( 2.79 ) |
| Parkinsonism | 15 | 74.67 ( 44.2 - 126.16 ) | 74.29 ( 1015.06 ) | 69.59 ( 41.19 ) | 6.12 ( 2.97 ) |
| Unresponsive To Stimuli | 15 | 5.76 ( 3.46 - 9.58 ) | 5.74 ( 58.41 ) | 5.71 ( 3.43 ) | 2.51 ( 1.42 ) |
| Opisthotonus | 14 | 48.59 ( 28.41 - 83.1 ) | 48.36 ( 621.65 ) | 46.34 ( 27.09 ) | 5.53 ( 2.76 ) |
| Hyperprolactinaemia | 14 | 12.46 ( 7.35 - 21.13 ) | 12.41 ( 145.2 ) | 12.28 ( 7.24 ) | 3.62 ( 2.06 ) |
| Delirium | 12 | 8.69 ( 4.92 - 15.35 ) | 8.65 ( 80.63 ) | 8.59 ( 4.86 ) | 3.1 ( 1.63 ) |
| Cogwheel Rigidity | 12 | 176.62 ( 95.86 - 325.4 ) | 175.88 ( 1795.51 ) | 151.48 ( 82.22 ) | 7.24 ( 2.73 ) |
| Urinary Retention | 12 | 9.47 ( 5.36 - 16.74 ) | 9.44 ( 89.79 ) | 9.37 ( 5.3 ) | 3.23 ( 1.7 ) |
| Blood Creatine Phosphokinase Increased | 12 | 7.52 ( 4.26 - 13.28 ) | 7.49 ( 67.09 ) | 7.45 ( 4.22 ) | 2.9 ( 1.51 ) |
| Tic | 10 | 3.78 ( 2.03 - 7.04 ) | 3.77 ( 20.32 ) | 3.76 ( 2.02 ) | 1.91 ( 0.71 ) |
| Sopor | 10 | 13.02 ( 6.97 - 24.31 ) | 12.97 ( 109.23 ) | 12.83 ( 6.87 ) | 3.68 ( 1.75 ) |
| Bradyphrenia | 10 | 41.86 ( 22.23 - 78.8 ) | 41.71 ( 382.7 ) | 40.21 ( 21.36 ) | 5.33 ( 2.25 ) |
| Eye Movement Disorder | 10 | 7.41 ( 3.97 - 13.81 ) | 7.38 ( 54.84 ) | 7.34 ( 3.94 ) | 2.88 ( 1.34 ) |
| Encephalitis Autoimmune | 10 | 49.47 ( 26.22 - 93.33 ) | 49.3 ( 452.66 ) | 47.2 ( 25.02 ) | 5.56 ( 2.29 ) |
| Cognitive Disorder | 10 | 7.29 ( 3.91 - 13.59 ) | 7.26 ( 53.69 ) | 7.22 ( 3.87 ) | 2.85 ( 1.33 ) |
| Urinary Tract Obstruction | 10 | 93.02 ( 48.72 - 177.62 ) | 92.7 ( 835.72 ) | 85.48 ( 44.77 ) | 6.42 ( 2.39 ) |
| Drug Withdrawal Syndrome Neonatal | 10 | 3.85 ( 2.07 - 7.17 ) | 3.84 ( 20.97 ) | 3.83 ( 2.06 ) | 1.94 ( 0.73 ) |
| Poisoning | 9 | 10.27 ( 5.32 - 19.82 ) | 10.24 ( 74.38 ) | 10.16 ( 5.26 ) | 3.34 ( 1.49 ) |
| Speech Disorder | 9 | 4.08 ( 2.12 - 7.87 ) | 4.07 ( 20.81 ) | 4.06 ( 2.11 ) | 2.02 ( 0.72 ) |
| Psychotic Disorder | 9 | 4.83 ( 2.51 - 9.3 ) | 4.82 ( 27.11 ) | 4.8 ( 2.49 ) | 2.26 ( 0.88 ) |
| Muscle Contractions Involuntary | 9 | 43.91 ( 22.53 - 85.58 ) | 43.77 ( 361.59 ) | 42.11 ( 21.61 ) | 5.4 ( 2.11 ) |
| White Blood Cell Count Increased | 9 | 6.18 ( 3.21 - 11.92 ) | 6.17 ( 38.76 ) | 6.14 ( 3.18 ) | 2.62 ( 1.1 ) |

Abbreviation: ROR, reporting odds ratio; PRR, proportional reporting ratio; EBGM, empirical Bayesian geometric mean; EBGM05, the lower limit of the 95% CI of EBGM; IC, information component; IC025, the lower limit of the 95% CI of the IC; CI, confidence interval; PT, preferred term.

**Supplementary Table 6**. Adverse events at the PT level for haloperidol in patients aged 18 to 65 from FAERS data.

| PT | Case numbers | ROR(95%CI) | PRR(χ2) | EBGM(EBGM05) | IC(IC025) |
| --- | --- | --- | --- | --- | --- |
| Weight Increased | 756 | 8.32 ( 7.74 - 8.95 ) | 8.09 ( 4689.26 ) | 8.05 ( 7.49 ) | 3.01 ( 2.89 ) |
| Extrapyramidal Disorder | 714 | 152.91 ( 141.37 - 165.39 ) | 148.43 ( 93896.38 ) | 133.37 ( 123.31 ) | 7.06 ( 6.7 ) |
| Neuroleptic Malignant Syndrome | 536 | 85.99 ( 78.73 - 93.93 ) | 84.11 ( 41364.21 ) | 79.08 ( 72.4 ) | 6.31 ( 5.98 ) |
| Intentional Self-Injury | 339 | 27.12 ( 24.34 - 30.22 ) | 26.75 ( 8239.61 ) | 26.24 ( 23.54 ) | 4.71 ( 4.45 ) |
| Dystonia | 310 | 58.12 ( 51.84 - 65.17 ) | 57.39 ( 16457.41 ) | 55.02 ( 49.07 ) | 5.78 ( 5.38 ) |
| Intentional Overdose | 284 | 7.41 ( 6.59 - 8.33 ) | 7.33 ( 1546.67 ) | 7.3 ( 6.49 ) | 2.87 ( 2.66 ) |
| Drug Interaction | 262 | 4.15 ( 3.68 - 4.69 ) | 4.12 ( 618.82 ) | 4.11 ( 3.64 ) | 2.04 ( 1.84 ) |
| Tremor | 259 | 3.84 ( 3.4 - 4.34 ) | 3.81 ( 537.18 ) | 3.8 ( 3.36 ) | 1.93 ( 1.73 ) |
| Somnolence | 222 | 2.83 ( 2.48 - 3.23 ) | 2.81 ( 259.64 ) | 2.81 ( 2.46 ) | 1.49 ( 1.28 ) |
| Drug Abuse | 199 | 3.4 ( 2.96 - 3.91 ) | 3.38 ( 333.65 ) | 3.37 ( 2.93 ) | 1.75 ( 1.53 ) |
| Agitation | 199 | 6.23 ( 5.42 - 7.17 ) | 6.19 ( 862.81 ) | 6.16 ( 5.36 ) | 2.62 ( 2.38 ) |
| Akathisia | 180 | 30.18 ( 26.02 - 35 ) | 29.96 ( 4927.31 ) | 29.31 ( 25.27 ) | 4.87 ( 4.45 ) |
| Dyskinesia | 174 | 12.44 ( 10.71 - 14.45 ) | 12.35 ( 1799.57 ) | 12.25 ( 10.54 ) | 3.61 ( 3.3 ) |
| Tachycardia | 172 | 3.87 ( 3.33 - 4.5 ) | 3.85 ( 362.22 ) | 3.84 ( 3.3 ) | 1.94 ( 1.7 ) |
| Muscle Rigidity | 169 | 34.25 ( 29.39 - 39.93 ) | 34.02 ( 5280.01 ) | 33.18 ( 28.47 ) | 5.05 ( 4.58 ) |
| Sopor | 164 | 19.37 ( 16.6 - 22.62 ) | 19.25 ( 2797.32 ) | 18.99 ( 16.26 ) | 4.25 ( 3.87 ) |
| Rhabdomyolysis | 159 | 8.88 ( 7.59 - 10.38 ) | 8.83 ( 1097.15 ) | 8.78 ( 7.5 ) | 3.13 ( 2.84 ) |
| Electrocardiogram Qt Prolonged | 153 | 9.44 ( 8.05 - 11.08 ) | 9.39 ( 1139.57 ) | 9.33 ( 7.95 ) | 3.22 ( 2.91 ) |
| Dysphagia | 151 | 4.55 ( 3.87 - 5.34 ) | 4.52 ( 413.73 ) | 4.51 ( 3.84 ) | 2.17 ( 1.91 ) |
| Catatonia | 150 | 60.34 ( 51.21 - 71.11 ) | 59.98 ( 8317.52 ) | 57.38 ( 48.7 ) | 5.84 ( 5.14 ) |
| Tardive Dyskinesia | 149 | 32.31 ( 27.45 - 38.03 ) | 32.12 ( 4384.86 ) | 31.37 ( 26.65 ) | 4.97 ( 4.47 ) |
| Self-Destructive Behaviour | 141 | 75.06 ( 63.31 - 88.99 ) | 74.63 ( 9689.19 ) | 70.65 ( 59.59 ) | 6.14 ( 5.32 ) |
| Suicide Attempt | 139 | 4.23 ( 3.58 - 5 ) | 4.21 ( 339.48 ) | 4.2 ( 3.55 ) | 2.07 ( 1.79 ) |
| Cognitive Disorder | 132 | 7.64 ( 6.43 - 9.07 ) | 7.6 ( 752.96 ) | 7.56 ( 6.37 ) | 2.92 ( 2.6 ) |
| Musculoskeletal Stiffness | 131 | 3.14 ( 2.65 - 3.73 ) | 3.13 ( 189.97 ) | 3.13 ( 2.63 ) | 1.64 ( 1.37 ) |
| Pulmonary Embolism | 128 | 3.07 ( 2.58 - 3.66 ) | 3.06 ( 177.75 ) | 3.06 ( 2.57 ) | 1.61 ( 1.33 ) |
| Psychotic Disorder | 125 | 8.62 ( 7.23 - 10.28 ) | 8.58 ( 832.36 ) | 8.53 ( 7.15 ) | 3.09 ( 2.75 ) |
| Blood Creatine Phosphokinase Increased | 122 | 9.13 ( 7.63 - 10.91 ) | 9.08 ( 872.17 ) | 9.03 ( 7.55 ) | 3.17 ( 2.82 ) |
| Coma | 120 | 4.81 ( 4.02 - 5.75 ) | 4.79 ( 358.78 ) | 4.77 ( 3.99 ) | 2.26 ( 1.95 ) |
| Aggression | 120 | 6.11 ( 5.1 - 7.31 ) | 6.08 ( 507.56 ) | 6.06 ( 5.06 ) | 2.6 ( 2.28 ) |
| Schizophrenia | 118 | 18.36 ( 15.3 - 22.02 ) | 18.27 ( 1900.36 ) | 18.03 ( 15.03 ) | 4.17 ( 3.71 ) |
| Cardiac Arrest | 112 | 2.87 ( 2.38 - 3.46 ) | 2.86 ( 135.64 ) | 2.86 ( 2.37 ) | 1.52 ( 1.22 ) |
| Parkinsonism | 112 | 36.84 ( 30.52 - 44.47 ) | 36.68 ( 3781.18 ) | 35.7 ( 29.58 ) | 5.16 ( 4.5 ) |
| Embolism Venous | 98 | 90.66 ( 73.85 - 111.3 ) | 90.3 ( 8094.3 ) | 84.52 ( 68.84 ) | 6.4 ( 5.22 ) |
| Sedation | 93 | 7.87 ( 6.41 - 9.65 ) | 7.84 ( 552.05 ) | 7.8 ( 6.36 ) | 2.96 ( 2.56 ) |
| Dysarthria | 90 | 5.18 ( 4.21 - 6.38 ) | 5.17 ( 301.65 ) | 5.15 ( 4.19 ) | 2.37 ( 2 ) |
| Delirium | 89 | 8.17 ( 6.63 - 10.06 ) | 8.14 ( 554.29 ) | 8.1 ( 6.57 ) | 3.02 ( 2.6 ) |
| Speech Disorder | 87 | 4.17 ( 3.38 - 5.15 ) | 4.16 ( 208.24 ) | 4.15 ( 3.36 ) | 2.05 ( 1.69 ) |
| Abnormal Behaviour | 86 | 6.53 ( 5.28 - 8.08 ) | 6.51 ( 399.69 ) | 6.49 ( 5.25 ) | 2.7 ( 2.3 ) |
| Hypertonia | 85 | 44.8 ( 36.08 - 55.63 ) | 44.64 ( 3507.01 ) | 43.2 ( 34.79 ) | 5.43 ( 4.54 ) |
| Torsade De Pointes | 85 | 23.76 ( 19.16 - 29.45 ) | 23.68 ( 1813.42 ) | 23.27 ( 18.77 ) | 4.54 ( 3.89 ) |
| Hallucination | 83 | 4.1 ( 3.31 - 5.09 ) | 4.09 ( 193.58 ) | 4.08 ( 3.29 ) | 2.03 ( 1.66 ) |
| Sluggishness | 76 | 17.06 ( 13.6 - 21.39 ) | 17 ( 1130.33 ) | 16.8 ( 13.39 ) | 4.07 ( 3.47 ) |
| Salivary Hypersecretion | 74 | 16.33 ( 12.98 - 20.55 ) | 16.28 ( 1048.66 ) | 16.1 ( 12.79 ) | 4.01 ( 3.41 ) |
| Restlessness | 72 | 4.32 ( 3.43 - 5.45 ) | 4.31 ( 182.82 ) | 4.3 ( 3.41 ) | 2.11 ( 1.7 ) |
| Drooling | 71 | 30.31 ( 23.95 - 38.36 ) | 30.22 ( 1960.93 ) | 29.56 ( 23.36 ) | 4.89 ( 4.06 ) |
| Swollen Tongue | 67 | 4.86 ( 3.82 - 6.18 ) | 4.85 ( 203.98 ) | 4.83 ( 3.8 ) | 2.27 ( 1.84 ) |
| Hyperprolactinaemia | 67 | 30.05 ( 23.58 - 38.29 ) | 29.97 ( 1834.08 ) | 29.32 ( 23 ) | 4.87 ( 4.02 ) |
| Depressed Level Of Consciousness | 67 | 3.83 ( 3.01 - 4.86 ) | 3.82 ( 139.03 ) | 3.81 ( 3 ) | 1.93 ( 1.52 ) |
| Pneumonia Aspiration | 66 | 7.73 ( 6.07 - 9.85 ) | 7.71 ( 383.49 ) | 7.67 ( 6.02 ) | 2.94 ( 2.45 ) |

Abbreviation: ROR, reporting odds ratio; PRR, proportional reporting ratio; EBGM, empirical Bayesian geometric mean; EBGM05, the lower limit of the 95% CI of EBGM; IC, information component; IC025, the lower limit of the 95% CI of the IC; CI, confidence interval; PT, preferred term.

**Supplementary Table 7**. Adverse events at the PT level for haloperidol in patients aged over 65 from FAERS data.

| PT | Case numbers | ROR(95%CI) | PRR(χ2) | EBGM(EBGM05) | IC(IC025) |
| --- | --- | --- | --- | --- | --- |
| Drug Interaction | 143 | 4.92 ( 4.17 - 5.81 ) | 4.85 ( 438.01 ) | 4.84 ( 4.1 ) | 2.28 ( 1.99 ) |
| Confusional State | 127 | 4.06 ( 3.4 - 4.83 ) | 4.01 ( 287.06 ) | 4 ( 3.36 ) | 2 ( 1.71 ) |
| Neuroleptic Malignant Syndrome | 123 | 114.48 ( 95.41 - 137.36 ) | 112.68 ( 13004.48 ) | 107.66 ( 89.72 ) | 6.75 ( 5.59 ) |
| Extrapyramidal Disorder | 119 | 103.05 ( 85.66 - 123.96 ) | 101.48 ( 11359.63 ) | 97.39 ( 80.96 ) | 6.61 ( 5.48 ) |
| Somnolence | 118 | 4.85 ( 4.05 - 5.82 ) | 4.8 ( 354.88 ) | 4.79 ( 3.99 ) | 2.26 ( 1.95 ) |
| Tremor | 96 | 4.31 ( 3.52 - 5.27 ) | 4.27 ( 240.57 ) | 4.26 ( 3.49 ) | 2.09 ( 1.75 ) |
| Delirium | 89 | 11.48 ( 9.31 - 14.16 ) | 11.36 ( 838.11 ) | 11.32 ( 9.18 ) | 3.5 ( 3.04 ) |
| Muscle Rigidity | 87 | 50.54 ( 40.83 - 62.57 ) | 49.99 ( 4092.31 ) | 48.99 ( 39.57 ) | 5.61 ( 4.67 ) |
| Agitation | 84 | 11.2 ( 9.03 - 13.9 ) | 11.09 ( 768.69 ) | 11.05 ( 8.91 ) | 3.47 ( 2.99 ) |
| Sopor | 82 | 31.4 ( 25.23 - 39.09 ) | 31.08 ( 2357.63 ) | 30.7 ( 24.66 ) | 4.94 ( 4.18 ) |
| Coma | 72 | 12.85 ( 10.19 - 16.22 ) | 12.74 ( 775.66 ) | 12.68 ( 10.05 ) | 3.66 ( 3.11 ) |
| Dysphagia | 70 | 4.53 ( 3.58 - 5.73 ) | 4.5 ( 190.4 ) | 4.49 ( 3.55 ) | 2.17 ( 1.75 ) |
| Parkinsonism | 70 | 39.09 ( 30.83 - 49.55 ) | 38.74 ( 2533.4 ) | 38.14 ( 30.09 ) | 5.25 ( 4.3 ) |
| Electrocardiogram Qt Prolonged | 65 | 12.55 ( 9.82 - 16.03 ) | 12.45 ( 681.55 ) | 12.39 ( 9.7 ) | 3.63 ( 3.04 ) |
| Hallucination | 62 | 4.81 ( 3.74 - 6.18 ) | 4.78 ( 185.18 ) | 4.77 ( 3.71 ) | 2.25 ( 1.8 ) |
| Tachycardia | 54 | 5.77 ( 4.41 - 7.54 ) | 5.73 ( 210.74 ) | 5.72 ( 4.38 ) | 2.52 ( 2.01 ) |
| Depressed Level Of Consciousness | 54 | 8.46 ( 6.47 - 11.06 ) | 8.4 ( 351.33 ) | 8.38 ( 6.41 ) | 3.07 ( 2.49 ) |
| Catatonia | 53 | 105.3 ( 79.91 - 138.77 ) | 104.59 ( 5210.52 ) | 100.25 ( 76.08 ) | 6.65 ( 4.74 ) |
| Pneumonia Aspiration | 50 | 8.58 ( 6.49 - 11.33 ) | 8.53 ( 331.35 ) | 8.5 ( 6.43 ) | 3.09 ( 2.48 ) |
| Musculoskeletal Stiffness | 49 | 5.14 ( 3.88 - 6.8 ) | 5.11 ( 161.84 ) | 5.1 ( 3.85 ) | 2.35 ( 1.83 ) |
| Hyponatraemia | 48 | 2.99 ( 2.25 - 3.97 ) | 2.97 ( 62.97 ) | 2.97 ( 2.24 ) | 1.57 ( 1.1 ) |
| Cardiac Arrest | 45 | 3.51 ( 2.62 - 4.7 ) | 3.49 ( 80.14 ) | 3.49 ( 2.6 ) | 1.8 ( 1.3 ) |
| Sedation | 43 | 18.41 ( 13.62 - 24.87 ) | 18.31 ( 698.49 ) | 18.18 ( 13.45 ) | 4.18 ( 3.27 ) |
| Dyskinesia | 41 | 7.92 ( 5.83 - 10.77 ) | 7.88 ( 245.84 ) | 7.86 ( 5.78 ) | 2.97 ( 2.31 ) |
| Overdose | 40 | 3.27 ( 2.39 - 4.46 ) | 3.25 ( 62.5 ) | 3.25 ( 2.38 ) | 1.7 ( 1.17 ) |
| Dysarthria | 39 | 7.21 ( 5.26 - 9.88 ) | 7.18 ( 207.02 ) | 7.16 ( 5.23 ) | 2.84 ( 2.18 ) |
| Speech Disorder | 39 | 5.27 ( 3.85 - 7.22 ) | 5.25 ( 134.04 ) | 5.24 ( 3.83 ) | 2.39 ( 1.79 ) |
| Rhabdomyolysis | 38 | 5.3 ( 3.85 - 7.29 ) | 5.28 ( 131.6 ) | 5.27 ( 3.83 ) | 2.4 ( 1.78 ) |
| Hypothermia | 38 | 25.21 ( 18.3 - 34.73 ) | 25.09 ( 870.1 ) | 24.84 ( 18.03 ) | 4.63 ( 3.48 ) |
| Tardive Dyskinesia | 38 | 41.95 ( 30.41 - 57.85 ) | 41.74 ( 1485.49 ) | 41.05 ( 29.76 ) | 5.36 ( 3.87 ) |
| Bradykinesia | 35 | 32.53 ( 23.29 - 45.44 ) | 32.39 ( 1050.51 ) | 31.97 ( 22.88 ) | 5 ( 3.62 ) |
| Cognitive Disorder | 35 | 5.82 ( 4.17 - 8.11 ) | 5.8 ( 138.64 ) | 5.78 ( 4.15 ) | 2.53 ( 1.87 ) |
| Dry Mouth | 34 | 2.89 ( 2.06 - 4.05 ) | 2.88 ( 41.8 ) | 2.88 ( 2.06 ) | 1.53 ( 0.96 ) |
| Gait Inability | 31 | 3.57 ( 2.51 - 5.09 ) | 3.56 ( 57.12 ) | 3.56 ( 2.5 ) | 1.83 ( 1.21 ) |
| Altered State Of Consciousness | 31 | 7.27 ( 5.11 - 10.36 ) | 7.25 ( 166.58 ) | 7.23 ( 5.08 ) | 2.85 ( 2.09 ) |
| Aggression | 30 | 8.24 ( 5.75 - 11.8 ) | 8.21 ( 189.36 ) | 8.18 ( 5.71 ) | 3.03 ( 2.21 ) |
| Blood Creatine Phosphokinase Increased | 30 | 7.3 ( 5.09 - 10.45 ) | 7.27 ( 161.86 ) | 7.25 ( 5.06 ) | 2.86 ( 2.07 ) |
| Dystonia | 29 | 35.26 ( 24.42 - 50.91 ) | 35.13 ( 947.81 ) | 34.64 ( 23.99 ) | 5.11 ( 3.5 ) |
| Urinary Retention | 29 | 4.39 ( 3.05 - 6.33 ) | 4.38 ( 75.57 ) | 4.37 ( 3.04 ) | 2.13 ( 1.45 ) |
| Parotitis | 28 | 159.4 ( 108.66 - 233.82 ) | 158.83 ( 4118.18 ) | 149.01 ( 101.58 ) | 7.22 ( 4.06 ) |
| Akathisia | 28 | 61.77 ( 42.42 - 89.94 ) | 61.55 ( 1626.07 ) | 60.03 ( 41.22 ) | 5.91 ( 3.76 ) |
| Unresponsive To Stimuli | 28 | 6.6 ( 4.55 - 9.57 ) | 6.58 ( 132.12 ) | 6.56 ( 4.53 ) | 2.71 ( 1.92 ) |
| Loss Of Personal Independence In Daily Activities | 27 | 3.41 ( 2.34 - 4.97 ) | 3.4 ( 45.72 ) | 3.4 ( 2.33 ) | 1.76 ( 1.1 ) |
| Restlessness | 26 | 6.25 ( 4.25 - 9.19 ) | 6.23 ( 113.95 ) | 6.22 ( 4.23 ) | 2.64 ( 1.82 ) |
| Bradyphrenia | 24 | 25.17 ( 16.83 - 37.66 ) | 25.1 ( 549.62 ) | 24.85 ( 16.61 ) | 4.64 ( 3.09 ) |
| Torsade De Pointes | 24 | 16.86 ( 11.28 - 25.2 ) | 16.81 ( 354.4 ) | 16.7 ( 11.17 ) | 4.06 ( 2.78 ) |
| Disorientation | 23 | 3.1 ( 2.06 - 4.67 ) | 3.1 ( 32.66 ) | 3.09 ( 2.05 ) | 1.63 ( 0.92 ) |
| Serotonin Syndrome | 23 | 12.9 ( 8.56 - 19.44 ) | 12.86 ( 250.36 ) | 12.8 ( 8.49 ) | 3.68 ( 2.51 ) |
| Intentional Self-Injury | 22 | 38.74 ( 25.41 - 59.07 ) | 38.64 ( 793.83 ) | 38.04 ( 24.95 ) | 5.25 ( 3.26 ) |
| Miosis | 21 | 24.41 ( 15.87 - 37.54 ) | 24.34 ( 465.39 ) | 24.11 ( 15.68 ) | 4.59 ( 2.94 ) |

Abbreviation: ROR, reporting odds ratio; PRR, proportional reporting ratio; EBGM, empirical Bayesian geometric mean; EBGM05, the lower limit of the 95% CI of EBGM; IC, information component; IC025, the lower limit of the 95% CI of the IC; CI, confidence interval; PT, preferred term.
